# A Multi-Strategy Improved Red-Billed Blue Magpie Optimizer for Global Optimization

**DOI:** 10.3390/biomimetics10090557

**Published:** 2025-08-22

**Authors:** Mingjun Ye, Xiong Wang, Zihao Guo, Bin Hu, Li Wang

**Affiliations:** 1 School of Information Science and Technology, Yunnan Normal University, Kunming 650000, China; 2 Southwest United Graduate School, Kunming 650000, China; 3 School of Information Science and Engineering, Yunnan University, Kunming 650000, China; 4 Télécom SudParis, Institut Polytechnique de Paris, 91120 Palaiseau, France; 5 Department of Computer Science and Technology, Kean University, Union, NJ 07083, USA; 6 School of Electrical and Electronic Information, Xihua University, Chengdu 610039, China

**Keywords:** red-billed blue magpie optimizer, swarm intelligence, boundary constraints, lévy flight, individual optimality

## Abstract

To enhance the convergence efficiency and solution precision of the Red-billed Blue Magpie Optimizer (RBMO), this study proposes a Multi-Strategy Enhanced Red-billed Blue Magpie Optimizer (MRBMO). The principal methodological innovations encompass three aspects: (1) Development of a novel dynamic boundary constraint handling mechanism that strengthens algorithmic exploration capabilities through adaptive regression strategy adjustment for boundary-transgressing particles; (2) Incorporation of an elite guidance strategy during the predation phase, establishing a guided search framework that integrates historical individual optimal information while employing a Lévy Flight strategy to modulate search step sizes, thereby achieving effective balance between global exploration and local exploitation capabilities; (3) Comprehensive experimental evaluations conducted on the CEC2017 and CEC2022 benchmark test suites demonstrate that MRBMO significantly outperforms classical enhanced algorithms and exhibits competitive performance against state-of-the-art optimizers across 41 standardized test functions. The practical efficacy of the algorithm is further validated through successful applications to four classical engineering design problems, confirming its robust problem-solving capabilities.

## 1. Introduction

Optimization problems are widespread [1], defined as the process of identifying the optimal solution from feasible solutions to minimize or maximize a given objective [2]. Optimization problems are generally characterized by large scale, numerous constraints, complex parameter control, and high computational cost [3]. In many optimization problems, it is necessary to find an optimal solution to a particular problem with highly complex constraints in a reasonable time period [4]. Effective methods are required to solve optimization problems involving numerous decision variables, complex nonlinear constraints, and objective functions [5]. Traditional optimization methods, represented by gradient descent and Lagrange multiplier methods, derive their efficacy strictly from mathematical properties, such as the differentiability and convexity of the objective function, along with the explicit formulation of constraint conditions [6]. However, optimization problems commonly encountered in real-world production and daily life are typically characterized by large-scale, high-dimensional, and nonlinear features [7], thus conventional methods often struggle to obtain acceptable solutions within a feasible time frame [8]. Specifically, the problem presents the following challenges:

- Multimodal: Many optimization problems exhibit multiple local optima, causing algorithms to easily become trapped in local optima and to fail to obtain satisfactory solutions.
- High-dimensional: As the number of decision variables increases, the problem’s dimensionality grows accordingly. The search space expands exponentially with increasing dimensions, leading to significantly higher problem complexity.
- Nonlinear: The objective functions of many problems are frequently nonlinear and may even be non-differentiable, rendering certain algorithms inapplicable due to their strict requirements on objective function properties.
- Multi-objective: Some problems require simultaneous optimization of multiple objectives that often exhibit conflicting relationships, making it difficult or impossible to find solutions that satisfy all objectives concurrently.

In contrast to conventional algorithms, heuristic algorithms [9] demonstrate the capability to identify a feasible suboptimal solution for optimization problems within a reasonable timeframe or under constrained computational resources. This methodology achieves computational feasibility and suboptimality through a systematic trade-off between solution accuracy/precision and operational efficiency [10]. However, these approaches frequently encounter convergence limitations toward local optima due to their relatively simplistic search patterns, which restrict their exploration capacity in complex solution spaces. The metaheuristic algorithm further combines the stochastic algorithm with the local search of the traditional heuristic algorithm to enhance the ability of the algorithm to jump out of the local optimal solution. It combines the laws of nature and biological laws for better solution results compared to heuristic algorithms [11].

Swarm Intelligence (SI) [12] is a significant branch of Artificial Intelligence (AI) that is grounded in the intelligent collective behavior observed in social groups in nature [13]. Researchers draw inspiration from various species and natural phenomena, leading to the development of various metaheuristic optimization algorithms based on SI [14]. The Particle Swarm Optimization Algorithm (PSO) [15] conceptualizes feasible solutions to optimization problems as particles within a search space. Each particle possesses a specific velocity and position, updating these attributes based on its historical optimal position and the best position identified within the group, thereby facilitating the search for superior solutions. In this context, each particle represents a candidate solution within the solution space. Ant Colony Optimization (ACO) [16] is another metaheuristic optimization algorithm inspired by the foraging behavior of natural ant colonies. Its fundamental principle involves addressing combinatorial optimization problems by simulating the collaborative behavior of ants as they release and perceive pheromones during food-searching activities. This bio-inspired algorithm, governed by simple rules that emulate biological swarm intelligence, provides an effective means of tackling complex optimization challenges. The Firefly Algorithm (FA) [17] is a metaheuristic driven by swarm intelligence inspired by the bioluminescent attraction mechanisms of fireflies. It utilizes brightness-mediated interactions to guide individuals toward optimal solutions, achieving a balance between global exploration and local exploitation through adaptive attraction dynamics and stochastic movement. Grey Wolf Optimization (GWO) [18] establishes an optimization search framework based on biological group intelligence by simulating the hierarchy, collaborative predation strategies, and group decision-making mechanisms of grey wolf populations in nature. The algorithm categorizes individual grey wolves into a four-level hierarchical structure with a strict social division of labor: α wolves serve as population leaders responsible for global decision-making; β wolves act as secondary coordinators assisting in decision execution; δ wolves form the base population unit and engage in local searches; and ω wolves function as followers, completing comprehensive explorations of the solution space. The Whale Optimization Algorithm (WOA) [19] mimics the feeding behavior of whales, particularly humpback whales, in the ocean, addressing complex optimization problems through strategies of distributed search, autonomous decision-making, and adaptive adjustment. The Sparrow Search Algorithm [20] simulates the role differentiation of sparrows during foraging, distinguishing between the leader (the sparrow that locates food) and the follower (the sparrow that trails the leader). The leader primarily focuses on finding food, while the follower remains within a certain range of the leader to assist in locating food. Additionally, sparrows make positional adjustments to evade predators, and these behavioral characteristics are abstracted into key steps within the algorithm to optimize the objective function.

However, as asserted by the “No Free Lunch” (NFL) theorem [21], every algorithm has inherent limitations, and no single algorithm can solve all optimization problems. Consequently, many researchers are dedicated to proposing new algorithms or enhancing existing ones. For instance, Zhu Fang et al. [22] introduced a good point set strategy during the initialization phase of the dung beetle optimization algorithm to increase population diversity. They also proposed a new nonlinear convergence factor to balance exploration and exploitation within the algorithm, as well as a dynamic balancing strategy between the number of dung beetles spawning and foraging. Additionally, they introduced a strategy based on perturbations from quantum computation and t-distributions to promote the algorithm’s ability of optimization search. Ya Shen et al. [23] proposed an improved whale optimization algorithm based on multiple population evolution to address the defects of the whale optimization algorithm, which has a slow convergence speed and easily falls into a local optimum. The population is further divided into three sub-populations based on the fitness value: exploratory population, exploitative population, and fitness population, and each sub-population performs a different updating strategy, and then it is experimentally verified that this multiple swarms co-evolutionary strategy can effectively enhance the algorithm’s optimization search capability. Yiying Zhang et al. [24] proposed an EJaya algorithm, whose local exploitation strategy enhances the local search capability by defining upper and lower bound local attraction points (combining the current optimal, worst, and population mean information), while the global exploration strategy expands the search scope by using random perturbations of historical population information to avoid stagnation at the later stage of the algorithm. Dhargupta et al. [25] proposed a Selective Opposition-based Grey Wolf Optimizer (SOGWO) that applies dimension-selective opposition learning to enhance exploration efficiency. By identifying ω-wolves through Spearman’s correlation analysis, the algorithm strategically targets low-status individuals for opposition learning, thereby reducing redundant search efforts and improving convergence speed while maintaining exploration–exploitation balance. Shuang Liang et al. [26] proposed an Enhanced Sparrow Search Swarm Optimizer (ESSSO); firstly, ESSSO introduces an adaptive sinusoidal walking strategy (SLM) based on the von Mises distribution, which enables individuals to dynamically adjust the learning rate to improve the evolutionary efficiency; secondly, a learning strategy with roulette wheel selection (LSR) is adopted to maintain the population diversity and prevent premature convergence; furthermore, a two-stage evolutionary strategy (TSE) is designed, which includes a mutation mechanism (AMM) to enhance the local searching ability and accelerate the algorithm’s convergence rate through a selection mechanism (SMS). Through the study, it is shown that the appropriately improved intelligent algorithm has better adaptability and effectiveness in some complex applications, and also has certain advantages compared with other heuristic algorithms.

The Red-billed Blue Magpie Optimizer (RBMO) [27] is a novel group intelligence-based metaheuristic algorithm inspired by the hunting behavior of red-billed blue magpies, which rely on community cooperation for activities such as searching for food, attacking prey, and food storage. The original paper applied the RBMO algorithm to various domains, including numerical optimization, engineering design problems, and UAV path planning. While the RBMO algorithm boasts a simple structure, minimal parameter requirements, and effective optimization performance, it still faces challenges in achieving optimal results for complex optimization problems. To address these challenges, we propose an improved version of the algorithm, termed the Multi-Strategy Enhanced Red-billed Blue Magpie Optimizer (MRBMO). This enhancement involves the design of a new boundary constraint and the establishment of an innovative prey attack model, which collectively improve the algorithm’s exploration and exploitation balance as well as its overall performance. The primary contributions of this paper are outlined as follows:

- A new boundary constraint processing method is designed and verified to accelerate the convergence speed and improve the exploitation ability of the algorithm. The method can also be used for other algorithms that requiring boundary processing.
- Inspired by particle swarm optimization (PSO), a new update method is redesigned in the development stage of the RBMO algorithm, which mainly introduces the optimal information of individual history to guide the algorithm to deeply explore the solution space. The step size is controlled by Lévy Flight to avoid the algorithm falling into the local optimal solution.
- The proposed MRBMO is evaluated on 41 benchmark functions. And its optimization performance is compared to eight other of the most advanced and high-performance algorithms. The MRBMO was successfully applied to four classical engineering design problems.

This paper is organized as follows. Section 2 introduces the foundational principles of the Red-billed Blue Magpie Optimization (RBMO) algorithm. In Section 3, we propose a multi-strategy enhanced version of the algorithm (MRBMO) to address the limitations of the original RBMO. Section 4 presents a comprehensive experimental comparison between MRBMO and eight other state-of-the-art optimization algorithms. To further validate the practical utility of the proposed improvements, Section 5 demonstrates the application of MRBMO in real-world engineering design problems. Finally, Section 6 summarizes the key contributions and findings of this study.

## 2. Red-Billed Blue Magpie Optimizer

In metaheuristic algorithms, exploration (diversification) and exploitation (intensification) work in tandem to determine the performance and efficiency of the algorithm [28]. On one hand, the algorithm must identify more promising regions by searching a broader space; on the other hand, it must also focus its resources on the in-depth development of these promising areas. Striking a balance between exploration and exploitation is often one of the primary challenges in the design of intelligent optimization algorithms [29]. An excessive inclination towards exploration can result in slow convergence and high computational costs, while an overemphasis on exploitation may cause the algorithm to prematurely converge to local optima, preventing it from discovering a globally optimal solution. In the Red-billed Blue Magpie Optimizer (RBMO), exploration and exploitation correspond to searching for prey and attacking prey, respectively.

### 2.1. Searching for Prey

When searching for prey, red-billed blue magpies usually operate in small groups (2–5 individuals) or collectively (more than 10 individuals) to exchange information. Therefore, two updating models were designed, with small groups and collective actions corresponding to Equations (1) and (2), respectively.

(1) $$X^{i}\left(t+1\right)=X^{i}\left(t\right)+\left(\frac{1}{p}\times \sum_{m=1}^{p}X^{m}\left(t\right)-X^{rs}\left(t\right)\right)\times Rand_{1}$$

(2) $$X^{i}\left(t+1\right)=X^{i}\left(t\right)+\left(\frac{1}{q}\times \sum_{m=1}^{q}X^{m}\left(t\right)-X^{rs}\left(t\right)\right)\times Rand_{2}$$

where *t* denotes the current iteration number, $X^{i}\left(t+1\right)$ denotes the *i*-th new search agent position, *p* and *q* denote the number of randomly selected individuals from the population, *p* ranges between 2 and 5, and *q* ranges between 10 and n, n is the size of the population, $X^{m}\left(t\right)$ denotes the *m*-th randomly selected individual, $X^{i}\left(t\right)$ denotes the *i*-th individual, and $X^{rs}\left(t\right)$ denotes the randomly selected search agent in the current iteration, the $Rand_{1}$ and $Rand_{2}$ denote two independent uniformly distributed random variables, each defined over the interval [0, 1).

### 2.2. Attacking the Prey

In small group operations, the main targets are small prey or plants. The corresponding mathematical model is shown in Equation (3). When acting in a collective manner, red-billed blue magpies are able to collectively target larger prey, such as large insects or small vertebrates. The mathematical representation of this behavior is shown in Equation (4).

(3) $$X^{i}\left(t+1\right)=X^{food}\left(t\right)+CF\times \left(\frac{1}{p}\times \sum_{m=1}^{p}X^{m}\left(t\right)-X^{i}\left(t\right)\right)\times Randn_{1}$$

(4) $$X^{i}\left(t+1\right)=X^{food}\left(t\right)+CF\times \left(\frac{1}{q}\times \sum_{m=1}^{q}X^{m}\left(t\right)-X^{i}\left(t\right)\right)\times Randn_{2}$$

where $X^{food}\left(t\right)$ represents the position of the food; it represents the solution with the minimum fitness value in the current iteration (i.e., the current best solution). $CF={\left(1-\left(\frac{t}{T}\right)\right)}^{\left(2\times \frac{t}{T}\right)}$ are coefficients that decrease nonlinearly with the number of iterations for adaptive control of the search step, T denotes the maximum number of iterations, and $Randn_{1}$ and $Randn_{2}$ represent a random number used to generate a standard normal distribution (mean 0, standard deviation 1).

### 2.3. Food Storage

In the original RBMO, the food storage behavior is defined as a greedy rule, and the new location is reserved only when the new generation fitness value is better, such as Equation (5).

(5) $$X^{i}\left(t+1\right)=\left\{\begin{array}{cc} X^{i}\left(t\right) & \mathrm{if}\;fitness_{old}^{i}>fitness_{new}^{i}\\ X^{i}\left(t+1\right) & else \end{array}\right.$$

where $fitness_{old}^{i}$ and $fitness_{new}^{i}$ represent the fitness values before and after position updating for the *i*-th Red-billed Blue Magpie, respectively.

### 2.4. Detailed Process of the RBMO

A control parameter ϵ=0.5 has been incorporated into the RBMO algorithm to enable adaptive selection between different update strategies. Specifically, the overall framework of the RBMO is shown as Algorithm 1.
**Algorithm 1:** The framework of the RBMO algorithm 
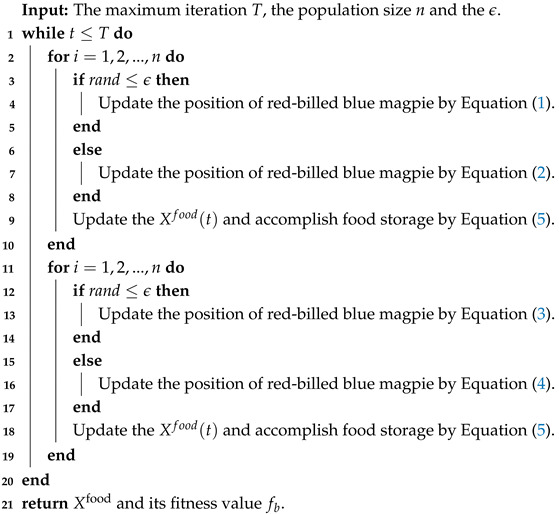



## 3. The Multi-Strategy Red-Billed Blue Magpie Optimizer

### 3.1. A New Way to Handle Boundary Constraints

In the original algorithm, when the dimension overstep occurs in the iterative process of the solution vector, the dimension overstep is usually assigned to the boundary in the way shown in Equation (6); this method is also used by most intelligent algorithms to transgress [30]. However, this method cannot effectively use historical search information, which can easily lead to premature loss of population diversity, which is not conducive to population convergence.

(6) $$X\left(i,d\right)=\left\{\begin{array}{cc} ub(d) & \;ub<X(i,d)\\ lb(d) & \;lb>X(i,d) \end{array}\right.$$

This study introduces a dynamic boundary correction strategy incorporating elite-guided dimensional adaptation. As formalized in Equation (7), the mechanism dynamically replaces out-of-bound dimension values with those from the global optimum’s corresponding dimensions. By leveraging dimensional information from elite individuals, this approach achieves three key advantages: (1) It establishes directional guidance through preferential dimension inheritance; (2) it fosters dimensional collaboration via cross-dimensional information exchange; and (3) it enhances exploitation capability through adaptive recombination of advantageous dimensional traits. The strategy effectively transforms isolated boundary handling into a coordinated optimization process, enabling more efficient exploitation of promising search regions while maintaining exploration diversity.

(7) $$X\left(i,d\right)=\left\{\begin{array}{cc} X^{food}\left(d\right) & ub<X(i,d)\;\mathrm{or}\;lb>X(i,d)\\ X(i,d) & \mathrm{otherwise} \end{array}\right.$$

Here, *d* represents the dimension index (d=1,2,…,D), where *D* is the total number of dimensions in the search space. ub(d) and lb(d) denote the upper and lower bounds of the *d*-th dimension, respectively, ensuring the solution remains within the feasible search region.

### 3.2. Individual Optimal Value Guidance

In the classical Particle Swarm Optimization (PSO) framework, the individual location updating mechanism integrates a dual guidance strategy that utilizes both the individual historical optimal solution (pbest) and the group historical optimal solution (gbest). This memory-driven approach effectively balances global exploration with local exploitation, providing a theoretical guarantee for algorithm convergence. However, in the original prey attack model of RBMO, the location update process only considered the one-way guidance of the global optimal solution. This limitation resulted in two significant drawbacks: (1) The neglect of individual cognitive experience led to a premature attenuation of population diversity; (2) The unipolar guidance mode is susceptible to causing search stagnation.

**Figure 1:**
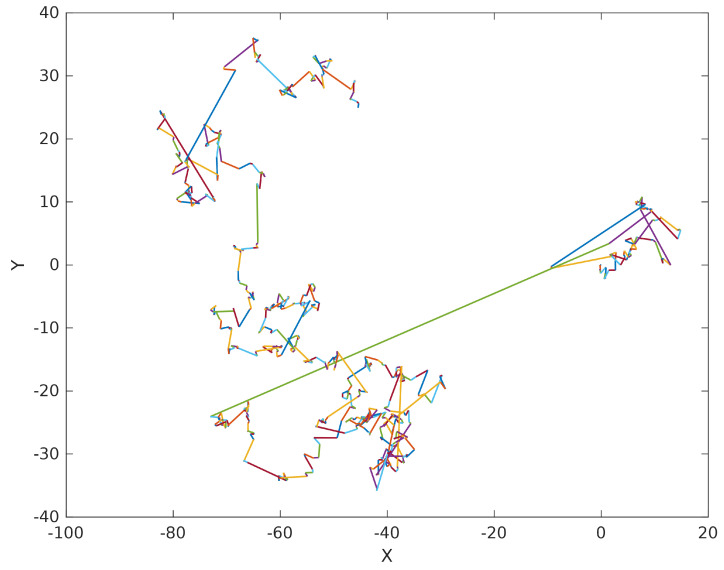
Lévy Flight Random Walk.

To address the aforementioned challenges, this study introduces an individual optimal value guidance strategy based on Lévy Flight [31] during the attack phase on prey. This strategy has dual optimization characteristics: first, the optimal solution for each individual by establishing a memory bank of individual optimal solutions; second, it employs the Lévy Flight distribution to generate the search step size. The power-law step size distribution, characterized by its heavy-tail property, effectively accommodates both local fine search and global mutation exploration. The combination of short-range, high-frequency jumps and long-range, low-frequency jumps produced by Lévy Flight significantly increases the probability of escaping local optimal while ensuring population diversity. In this study, the Mantegna algorithm was used to achieve efficient Lévy Flight simulation, and the step calculation process is shown in Equation (8).

(8) $$\left\{\begin{array}{c} u∼N(0,\sigma_{u}^{2}),\\ v∼N(0,\sigma_{v}^{2}),\\ \sigma_{v}=1,\\ \sigma_{u}={\left[\frac{\Gamma (1+\beta )\sin(\pi \beta /2)}{\Gamma \left(\frac{1+\beta }{2}\right)\beta \cdot 2^{(\beta -1)/2}}\right]}^{1/\beta },\\ s=\frac{u}{{\left|v\right|}^{1/\beta }}; \end{array}\right.$$

where β denotes the shape parameter, usually 0.3≤β≤1.99, Γ represents the gamma function, and *s* specifies the step size. Figure 1 illustrates the motion trajectory of Lévy Flight, whose random walk mechanism alternating between long and short step lengths can enhance the algorithm’s global search efficiency and its local optima avoidance capabilities [32]. The updated attack prey position is subsequently determined by Equations (9) and (10).

(9) $$X^{i}\left(t+1\right)=X^{food}\left(t\right)+CF\times \left(\frac{1}{p}\times \sum_{m=1}^{p}X^{m}\left(t\right)-X^{i}\left(t\right)\right)\times Randn_{1}+\left({pBest}^{\left(i\right)}\left(t\right)-X^{i}\left(t\right)\right)\times s$$

(10) $$X^{i}\left(t+1\right)=X^{food}\left(t\right)+CF\times \left(\frac{1}{q}\times \sum_{m=1}^{q}X^{m}\left(t\right)-X^{i}\left(t\right)\right)\times Randn_{2}+\left({pBest}^{\left(i\right)}\left(t\right)-X^{i}\left(t\right)\right)\times s$$

Here, ${pBest}^{\left(i\right)}\left(t\right)$ denotes the historical best value of the *i*-th individual at the *t*-th iteration, and its update rule is defined in Equation (11). It is worth noting that f(•) represents the objective function.

(11) $${pBest}^{\left(i\right)}\left(t\right)=\left\{\begin{array}{cc} X^{i}\left(t\right) & \mathrm{if}\;f\left(X^{i}\left(t\right)\right)<f\left({pBest}^{\left(i\right)}\left(t-1\right)\right)\\ {pBest}^{\left(i\right)}\left(t-1\right) & \mathrm{else} \end{array}\right.$$

### 3.3. Detailed Process of the MRBMO

The overall framework of the Multi-strategy Red-billed Blue Magpie Optimizer (MRBMO) algorithm is presented in Algorithm 2. Crucially, this enhanced implementation introduces no additional computational overhead compared to the baseline, thus preserving equivalent time complexity.
**Algorithm 2:** The framework of the MRBMO algorithm 
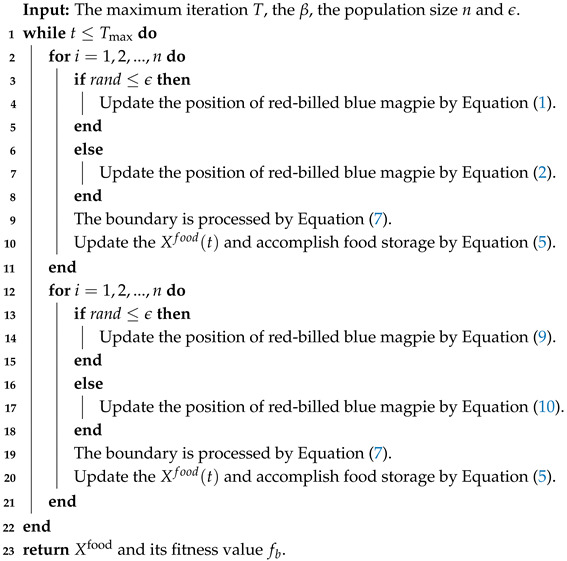



## 4. Numerical Experiment

The CEC2017 [33] test set is a set of benchmark functions for evaluating and comparing the performance of intelligent optimization algorithms. The test suite consists of 29 single-objective functions, covering a variety of complexities and diversity, designed to simulate real-world optimization problems and provide a fair evaluation platform for algorithmic researchers. This suite incorporates unimodal functions (F1–F2), which are characterized by a single global optimum devoid of local optima. These functions are primarily employed to evaluate the convergence velocity and exploration efficiency of optimization algorithms. Simple multimodal functions (F3–F9), containing multiple local optima, are used to evaluate the ability of the algorithm to avoid falling into local optima. Hybrid functions (F10–F19), which are composed of multiple basic functions, have a complex search space and test the performance of the algorithm when dealing with nonlinear and discontinuous problems. Composition functions (F20–F29), which are generated by multiple basic functions through rotation, migration and other operations, have higher complexity and are used to evaluate the performance of algorithms in high-dimensional and complex problems. The CEC2022 [34] benchmark function set is similar to CEC2017, with a total of 12 test functions: Unimodal functions, Basic functions, Hybrid functions, and Composition functions. CEC2022 is further optimized in terms of function complexity, diversity, and real-world problem simulation, enabling a more comprehensive evaluation of algorithm performance in different types of optimization problems. CEC2017 and CEC2022 test functions are detailed in Table A1 and Table A2.

### 4.1. Parameters Sensitivity Testing

In the stage of attacking prey, Lévy Flight is introduced as the step size. The parameter β of Lévy Flight is a constant, and its value significantly influences the performance of the algorithm. To select the parameters β and ϵ in the original algorithm, this paper designs a set of experiments for analysis. Specifically, we consider $\epsilon \in \left\{0.25,\;0.5,\;0.75\right\}$ and $\beta \in \left\{0.5,1,1.5\right\}$. The CEC2022 benchmark was utilized in the test set, with the dimension of the vector X set to 20; the maximum number of iterations was 500, the population size was 30, and the experiment was repeated 30 times. The experimental results are summarized in Table 1, which documents the average performance metrics and the corresponding rankings for each parameter configuration across 30 independent trials. To statistically evaluate the overall algorithmic performance, the Friedman rank sum test was applied to the ranked results, with lower average ranks indicating superior performance. In particular, the combination of parameters ϵ=0.75 and β=0.5 consistently achieved optimal performance on multiple benchmark functions. Based on these findings, this configuration is proposed as the recommended parameter setting for the proposed algorithm.

### 4.2. Ablation Experiment

To ascertain the impact of the enhanced strategy introduced in this paper on the performance of the algorithm, an ablation experiment is meticulously designed in this section. Building upon the foundational RBMO algorithm, the first modification involves refining the out-of-bounds processing method, hereafter referred to as RBMO1. Subsequently, the hunting behavior within the original RBMO algorithm is revised, and a novel update mechanism, designated as RBMO2, is conceptualized. These two enhancements are then integrated to form MRBMO, which represents the ultimate refined algorithm presented in this study. A comparative analysis of RBMO1, RBMO2, and MRBMO against the original RBMO algorithm is conducted to comprehensively assess the magnitude of the improvements achieved.

The CEC2022 benchmark suite was utilized to evaluate algorithm performance, with metrics including minimum and average values, standard deviations, and Friedman rankings calculated for each test function (Table 2). For the unimodal function F1, both RBMO1 and RBMO2 achieved superior values compared to RBMO, indicating that both strategies enhanced the performance of RBMO to varying degrees. A closer examination reveals that RBMO2 attained not only smaller values but also exhibited greater stability than RBMO1, suggesting that the second strategy contributed more significantly to the improvement of RBMO. For the hybrid function F6, analysis of average values reveals that implementing either strategy individually compromised the algorithm’s ability to escape local optima. However, their synergistic combination demonstrated superior performance, yielding solutions closest to the theoretical optimum. On composite functions F9–F12, the combination of the two strategies enhanced the convergence performance of RBMO. However, there remains a discrepancy from the theoretical optimum. Observing the final mean rank, both strategies enhance the overall performance of RBMO to varying degrees, with their synergistic combination yielding the most favorable outcomes.

### 4.3. Comparison of MRBMO with State-of-the-Art and Well-Established Algorithms

The comparison algorithms selected for this study encompass a diverse range of optimization techniques. These include the original Red-billed Blue Magpie Optimizer (RBMO), and two advanced algorithms: the Selective Opposition-based Grey Wolf Optimizer (SOGWO) [25] and the Transient Trigonometric Harris Hawks Optimizer (TTHHO) [35]. Additionally, the classical Particle Swarm Optimization (PSO) [15] is included, along with more recently developed methods, such as the Sparrow Search Algorithm (SSA) [20] and the RIME Optimization Algorithm (RIME) [36]. Top-performing algorithms from the CEC competitions LSHADE_SPACMA [37] are also incorporated. The parameter settings for each algorithm were strictly configured according to the recommendations provided in their respective original publications.

To further verify the effectiveness of the improved algorithm, the CEC2017 test function suite was used to fully verify the algorithm performance. First of all, the mean value, standard deviation, and ranking of the MRBMO and the comparison algorithm running 30 times with dimension 30, 50, and 100 were counted, respectively, as shown in Table 3, Table 4 and Table 5.

- When the dimension is 30, MRBMO achieved first-ranked average values for the unimodal functions F1 and F2. These results demonstrate the algorithm’s superior exploration capabilities. For simple multimodal functions, MRBMO achieved first-place average scores in F3–F9, outperforming all other algorithms. These results indicate that MRBMO possesses strong local-optimum avoidance capabilities. However, an examination of the standard deviation values reveals significant fluctuations in the results obtained by MRBMO. In Hybrid function F17, MRBMO (2.56E+03) is slightly inferior to RBMO (2.42E+03), but significantly superior to other algorithms. Looking at the standard deviation, it can be found that MRBMO has a lower standard deviation in most functions, and achieves the second or third standard deviation in very few functions. It shows that its performance is highly stable in different problems with little fluctuation, strong adaptability to problem characteristics, and better robustness.
- Furthermore, we investigate the impact of dimensionality changes on the performance of MRBMO. Notably, in experiments with 50 and 100 dimensions, MRBMO consistently maintains a stable first-place average ranking across most test functions. This indicates that as the dimensionality increases, its search performance does not deteriorate. Collectively, MRBMO remains highly competitive in addressing complex high-dimensional optimization challenges.

**Figure 2:**
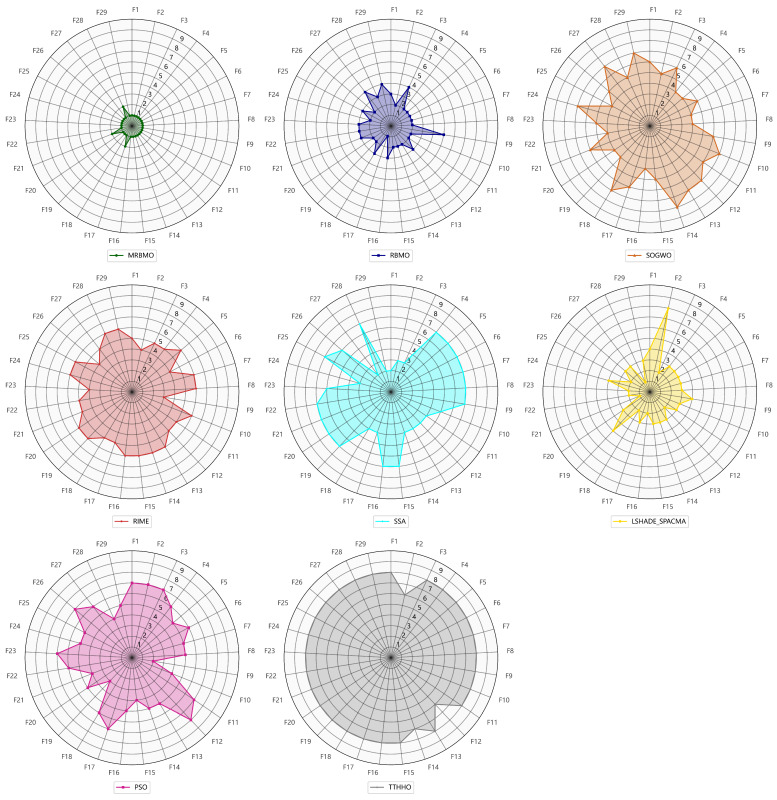
The radar of all algorithms.

A statistical analysis was conducted on the rankings of each algorithm across 29 benchmark functions in 30 dimensions. As illustrated in Figure 2, the MRBMO algorithm demonstrates superior performance, securing first place on 26 test functions while attaining second place on three functions (F17, F21 and F28). This dominance can be attributed to its multi-strategy framework, which effectively balances exploration and exploitation through dynamic boundary constraint handling and elite-guided Lévy Flight mechanisms. In contrast, other algorithms, such as SSA and PSO, exhibit significant performance fluctuations, demonstrating competitive results on specific functions while showing limited effectiveness on others.

**Figure 3:**
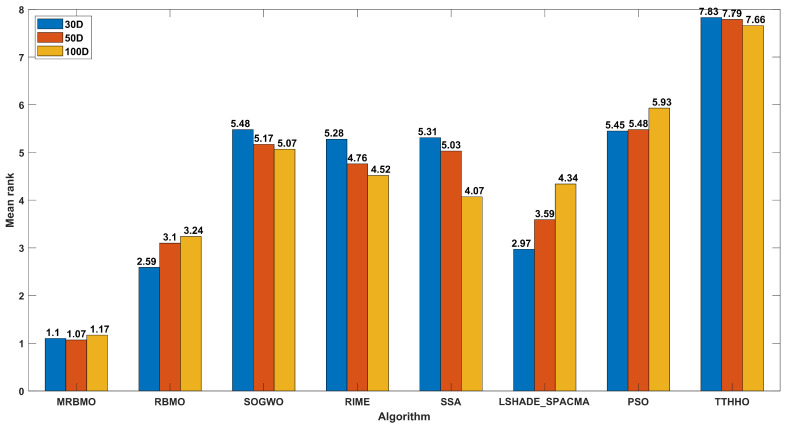
The results of the Friedman rank sum test of CEC2017.

Furthermore, to rigorously evaluate the comprehensive performance of all algorithms, the Friedman rank-sum test was employed. A bar chart depicting the mean ranks for all algorithms is presented in Figure 3, where lower numerical rankings indicate superior performance. The results demonstrate that across all tested dimensionalities, the MRBMO algorithm consistently attained the lowest average rank. This indicates the excellent robustness and insensitivity to dimension changes of MRBMO.

In this study, the Wilcoxon rank sum test [38], a nonparametric statistical method, was utilized to evaluate whether the performance differences between the improved method and the comparative algorithms are statistically significant. The test was conducted at a significance level of 0.05. A *p*-value lower than 0.05 indicates a significant difference between the two algorithms being compared, while a *p*-value of 0.05 or higher suggests that the performance of the two algorithms is not significantly different and can be considered comparable. The test results are presented in Table A3, Table A4 and Table A5, with data points exhibiting *p*-values greater than 0.05 highlighted in bold font. The experimental results are summarized in Table 6, where $R^{+}$ denotes the number of comparisons showing statistically significant differences, and $R^{-}$ represents the number of comparisons without statistically significant differences, with the final row presenting the aggregate results. It can be observed that the proposed method demonstrates statistically significant differences compared to competing algorithms in most test functions, and its superiority is substantiated.

### 4.4. Assessing Convergence Performance

**Figure 4:**
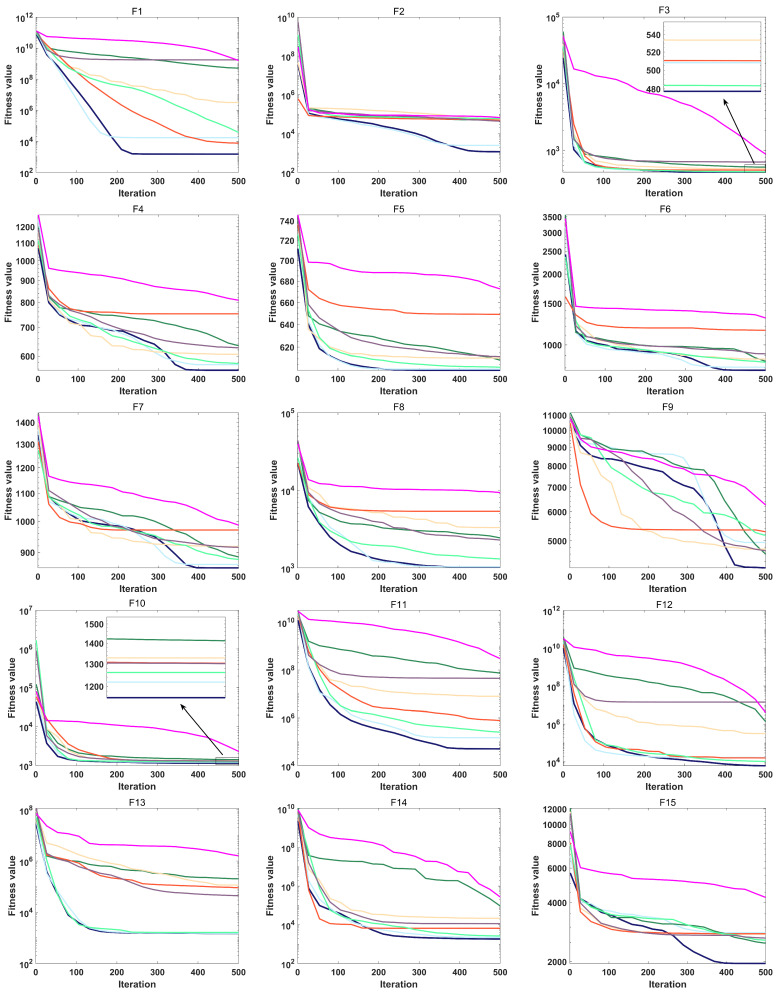
Convergence curve of F1–F15.

**Figure 5:**
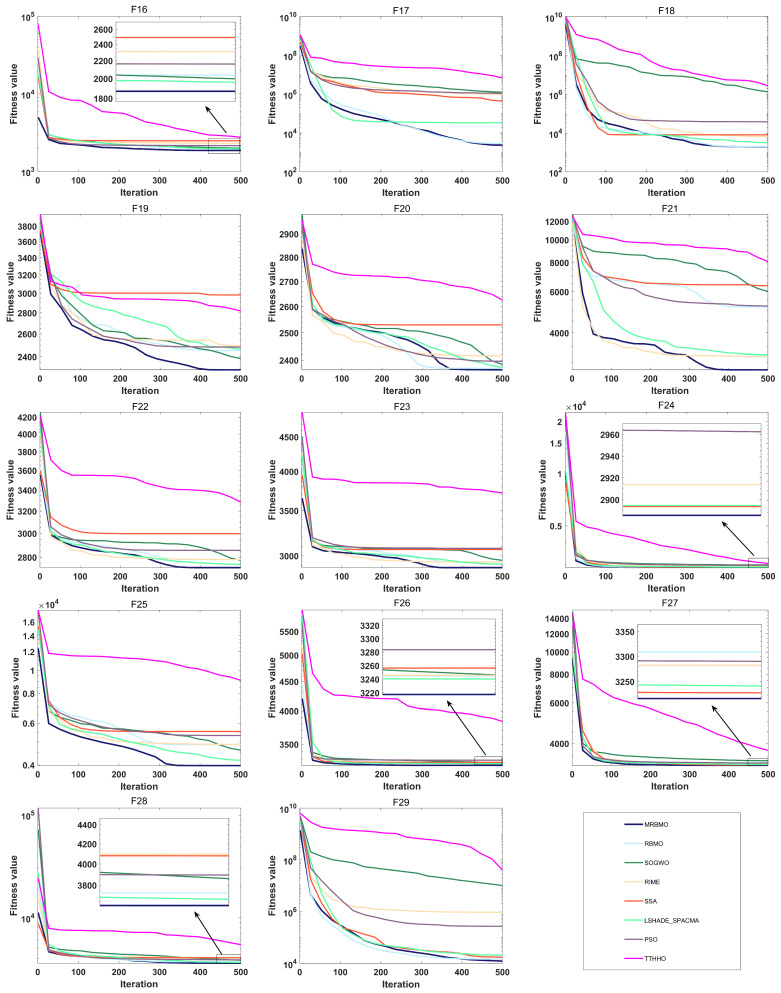
Convergence curve of F16–F29.

To assess both the accuracy and the convergence speed of the algorithms, convergence curves were plotted for MRBMO and the other algorithms at dimension 30, as illustrated in Figure 4 and Figure 5. It is worth noting that in each subplot, the horizontal axis represents the number of iterations, while the vertical axis represents the average convergence curve over 30 runs.

- During the optimization process of the unimodal test function F1, MRBMO exhibits faster convergence speed compared to other comparative algorithms. Notably, the MRBMO algorithm benefits from its enhanced global search capability, enabling it to obtain higher-quality feasible solutions. This advantageous characteristic is further validated in the optimization process of another unimodal function F2.
- During the optimization processes of simple multimodal functions F3 and F8, the MRBMO algorithm did not demonstrate significant competitive advantages. However, when addressing functions F4, F5, F6, and F7, MRBMO exhibited unique optimization characteristics as other comparative algorithms became trapped in local optima stagnation: Not only did the algorithm avoid convergence stagnation, but it also obtained superior solutions through significantly accelerated convergence, thereby validating the advancement of its local optima avoidance mechanism. Notably, in the optimization scenario of function F9, although MRBMO showed lower convergence rates than the SSA, RIME, and PSO algorithms during the initial phase (iteration count < 300), these contrast algorithms were constrained by premature convergence due to their inability to escape local extremum constraints. In stark contrast, MRBMO demonstrated stronger sustained optimization capability in later stages (iteration count > 300), eventually achieving gradual approximation to the global optimum. This phenomenon highlights the algorithm’s superiority in long-term convergence performance. Such phased performance disparities validate the design advantages of MRBMO in maintaining an exploration–exploitation balance, enabling it to demonstrate enhanced global convergence characteristics when addressing complex optimization problems.
- In the hybrid benchmark function tests, the MRBMO algorithm demonstrated significant performance advantages. Specifically, for the F11, F12, F14, F15, and F19 functions, this algorithm exhibited marked superiority over all reference algorithms in both key metrics: convergence rate and solution accuracy. Regarding the optimization of the F10 function, MRBMO showed the fastest convergence characteristics during the initial iteration phase (<100 iterations), with its solution quality rapidly approaching the theoretical optimum. During optimization processes for the F13, F17, and F18 functions, MRBMO achieved comparable convergence performance with the RBMO and LSHADE_SPACMA algorithms, with all three outperforming other comparative algorithms. Regarding the F16 function, in the early iteration stage, MRBMO merely maintained convergence levels comparable to those of other algorithms, without showcasing any remarkable superiority. In the later iteration stage, however, through continuous exploration, MRBMO managed to discover higher-quality solutions.
- The proposed MRBMO algorithm exhibits superior convergence rates for multiple composition functions. This is particularly evident in the test cases of F20, F21, F22, F23, F25, and F29. By contrast, for functions F24, F26, F27, and F28, the convergence performance of MRBMO is comparable to that of other comparative algorithms.

### 4.5. Population Diversity Analysis

Maintaining adequate population diversity throughout the optimization process is a critical factor influencing the performance of metaheuristic optimization algorithms, as highlighted in recent studies. Population diversity refers to the distribution of individuals within the search space and plays a pivotal role in balancing exploration and exploitation. When diversity is relatively high, individuals are more broadly distributed across the search space. This enhances the algorithm’s ability to explore various regions, thereby reducing the likelihood of becoming trapped in local optima. Conversely, lower levels of diversity may result in premature convergence, where the algorithm stagnates around suboptimal solutions due to insufficient exploration.

This section employs Equation (12) [39] to quantify population diversity. In this equation, the parameter $I_{C}\left(t\right)$ represents the degree of dispersion of the population relative to its center of mass during each iteration *t*. Specifically, $x_{id}\left(t\right)$ denotes the value of the *d*-th dimension of the *i*-th individual at iteration *t*. Here, n represents the population size, and D is the total number of dimensions in the search space. By calculating the distance of each individual from the center of mass, this metric provides a numerical measure of how dispersed the population is at any given point in the optimization process.

(12) $$\begin{array}{cc} \; & I_{C}\left(t\right)=\sqrt{\sum_{i=1}^{n}\sum_{d=1}^{D}{\left(x_{id}\left(t\right)-c_{d}\left(t\right)\right)}^{2}}\\ \; & c_{d}\left(t\right)=\frac{1}{D}\sum_{i=1}^{n}x_{id}\left(t\right) \end{array}$$

**Figure 6:**
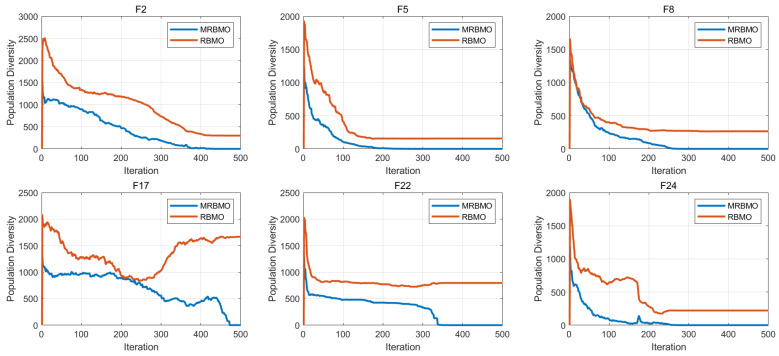
Population diversity analysis.

Figure 6 compares the population diversity dynamics of RBMO and MRBMO on CEC2017 benchmark functions on 30 dimensions. The figure reveals that population diversity generally exhibits a declining trend throughout the optimization process. This decline is expected, as the optimization progresses and the algorithm increasingly focuses on refining solutions near the optimal region. However, a notable difference emerges between RBMO and MRBMO: MRBMO demonstrates consistently lower diversity compared to RBMO. This phenomenon can be attributed to the boundary constraint strategy implemented in MRBMO. This strategy is designed to guide individuals toward feasible regions of the search space, potentially accelerating convergence toward the current optimal solution. While this approach enhances exploitation by focusing the search around promising areas, it simultaneously reduces the breadth of exploration.

### 4.6. Exploration and Exploitation Evaluation

**Figure 7:**
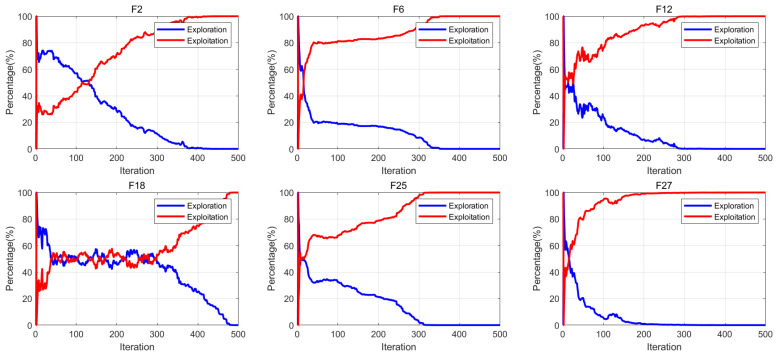
Exploration and exploitation evaluation.

During evolutionary optimization processes, metaheuristic algorithms employ distinct coordination mechanisms to balance exploration and exploitation among population members. This section uses a methodology [40] to quantify algorithmic search behavior through tracking dynamic variations in individual dimensional components, thereby enabling quantitative assessment of exploration–exploitation tendencies throughout the evolutionary process [41]. In this section, the exploration–exploitation balance of the algorithm is evaluated through the formulation defined in Equation (13). Where *X* denotes the population matrix at the *t*-th iteration (structured as an n × D matrix), Div(t) quantifies the dimensional diversity metric, $median\left(X_{d}\left(t\right)\right)$ corresponds to the median value of the *d*-th dimension across all individuals at iteration *t*, while $X_{id}\left(t\right)$ indicates the *d*-th dimensional value of the *i*-th individual. The exploration-to-exploitation ratio (ERP/ETP) corresponds to percentage-based metrics derived from these measurements. The experimental findings are comprehensively illustrated in Figure 7. It can be observed that as the iterations proceed, the algorithm’s exploitation curve progressively ascends towards 100%, while the exploration curve correspondingly diminishes to zero. This pattern indicates that the algorithm predominantly engages in exploratory behaviors during the initial stages, shifting its emphasis to exploitation in subsequent phases.

(13) $$\begin{array}{cc} \; & Div\left(t\right)=\frac{1}{D}\sum_{d=1}^{D}\frac{1}{n}\sum_{i=1}^{n}\left|median\left(X_{d}\left(t\right)\right)-X_{id}\left(t\right)\right|\\ \; & ERP=\frac{Div(t)}{\max(Div)}\times 100\%\\ \; & ETP=\frac{\left|\max(Div)-Div(t)\right|}{\max(Div)}\times 100\% \end{array}$$

## 5. Engineering Design Application

The engineering design optimization problem seeks to achieve optimal system performance through mathematical modeling and algorithmic solutions while satisfying physical constraints and performance metrics. To validate the efficacy of the proposed methodology, four engineering design problems were adopted in this section. For experimental rigor, identical parameters were configured: a population size of 30, a maximum iteration count of 100, and 30 independent trials to mitigate stochastic interference. Statistical metrics including the best value, mean, median, worst value, and standard deviation were systematically documented for each algorithm. Furthermore, convergence curves and box plots were generated to visualize the solution distributions and search efficiency across algorithmic approaches.

### 5.1. Extension/Compression Spring Design (TCSD)

**Figure 8:**
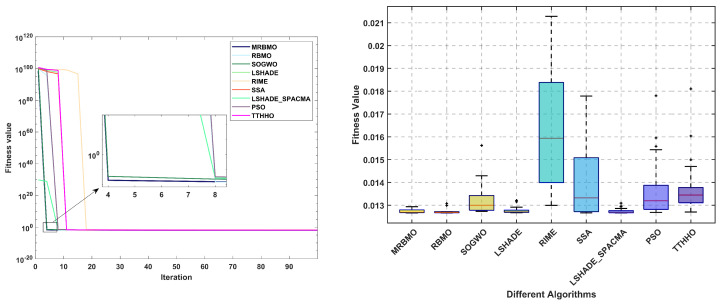
Curve and box plots of TCSD.

The extension/compression spring design problem [42], illustrated in Figure 8, seeks to minimize the spring weight by optimizing parameters such as the wire diameter (*d*), average coil diameter (*D*), and the number of active coils (*N*). This problem endeavors to identify the optimal parameter combination to achieve the desired performance while simultaneously minimizing the spring weight, thereby facilitating efficient and lightweight spring design for diverse applications. The mathematical model can be found in Appendix B.1.

The statistical results of 30 independent operations are presented in Table 7. It can be observed that, when compared with RMBO, the method proposed in this paper reveals improvements in the average value, the worst value, and the standard deviation. Nevertheless, it should not be overlooked that there still exist disparities with LSHADE_SPACMA and LSHADE in relation to different indicators. As demonstrated in Figure 8, during the initialization phase, all algorithms present relatively high fitness value distributions. As the iterations advance, they converge rapidly. A further analysis of the statistical characteristics of boxplots indicates that the MRBMO, RBMO, LSHADE, and LSHADE_SPACMA algorithms display a compact interquartile range (IQR), which affirms the strong stability of their optimization processes. Notably, MRBMO has the lowest occurrence of outliers, accentuating its superiority in solution quality control.

### 5.2. Reducer Design Problem (RDP)

**Figure 9:**
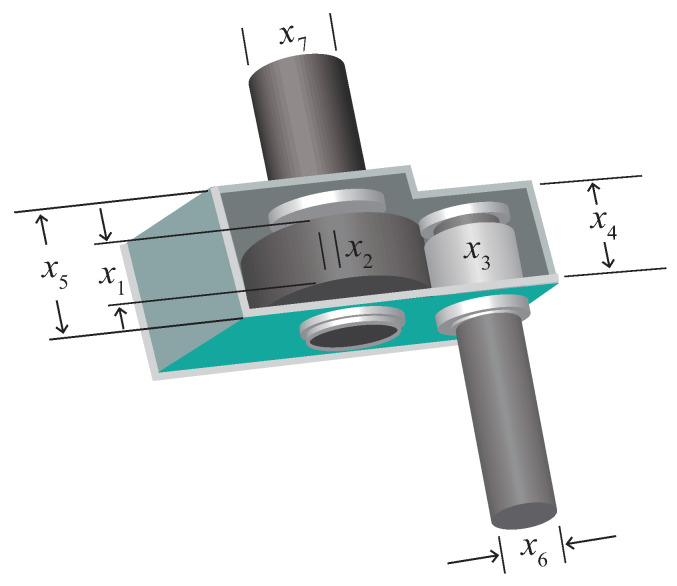
Structure diagram of RDP.

The schematic diagram of the speed reducer design problem [43] is depicted in Figure 9. The problem involves seven design variables: end face width ($x_{1}$), number of tooth modules ($x_{2}$), number of teeth in the pinion ($x_{3}$), length of the first shaft between the bearings ($x_{4}$), length of the second shaft between the bearings ($x_{5}$), diameter of the first shaft ($x_{6}$), and diameter of the second shaft ($x_{7}$). The objective of the problem is to minimize the total weight of the gearbox by optimizing seven variables. The mathematical model can be found in Appendix B.2.

**Figure 10:**
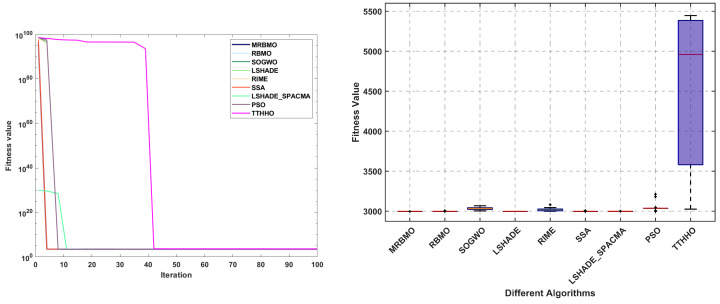
Curve and box plots of RDP.

Table 8 presents the statistical results, with the optimal outcomes highlighted in bold. It is evident that MRBMO achieves the most favorable results in terms of the best, mean, and median values, ranking second only to LSHADE in the worst and standard deviation categories. Subsequently, the convergence curves and box plots of the algorithms are illustrated in Figure 10. An analysis of the convergence curves reveals that, with the exception of the TTHHO algorithm—which exhibits a significantly slower convergence pace, all the compared algorithms demonstrate comparable convergence speeds. The box plots summarize the distribution of fitness values obtained from 30 independent runs for each algorithm, encompassing the median, quartiles, and outliers. The TTHHO algorithm features a substantially larger box plot accompanied by numerous outliers, indicating significant performance variability and multiple data points that deviate markedly from the central values. Conversely, the other algorithms exhibit more compact box plots, reflecting a higher degree of stability in their performance.

### 5.3. Welded Beam Design Problem (WBD)

**Figure 11:**
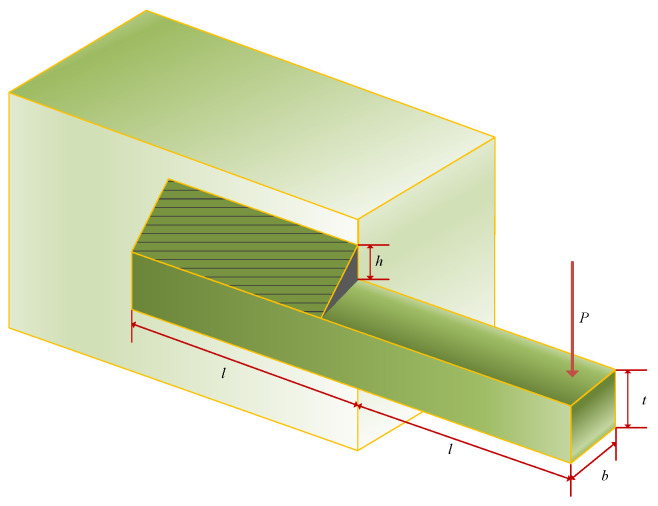
Structure diagram of WBD.

The objective of the welded beam design problem [44] is to minimize the cost of the welded beam. As shown in Figure 11, the welded beam design problem exists with four parametric variables: weld thickness (*h*), length of the connected portion of the bar (*l*), height of the bar (*t*), and thickness of the reinforcement bar (*b*). The mathematical model can be found in Appendix B.3.

**Figure 12:**
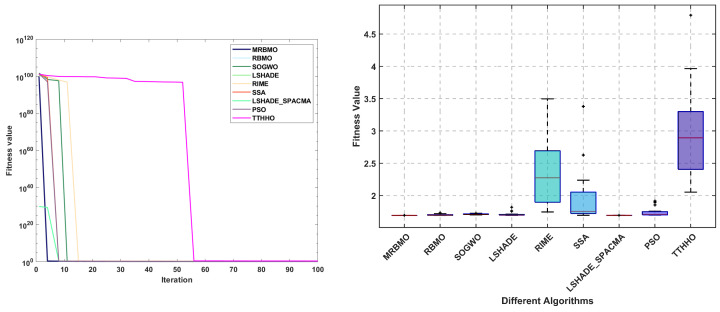
Curve and box plots of WBD.

The statistical results of 30 independent operations are shown in Table 9. It can be observed that MRBMO achieves optimal values in all statistical metrics, demonstrating excellent stability. Figure 12 presents the convergence curves and box plots of all algorithms. The analysis reveals that the fitness values of the cost function are relatively large during initialization, but most algorithms subsequently converge rapidly to superior values. In the box plots, MRBMO and LSHADE_SPACMA exhibit relatively shorter box lengths, indicating that the distribution of fitness values obtained from 30 independent runs is more concentrated, suggesting potentially higher algorithmic stability. Conversely, RIME, SSA and TTHHO display noticeable outliers (denoted by the ’+’ symbols), implying these algorithms may occasionally yield exceptionally superior or inferior fitness values under specific conditions.

### 5.4. Gear Train Design Problem (GTD)

**Figure 13:**
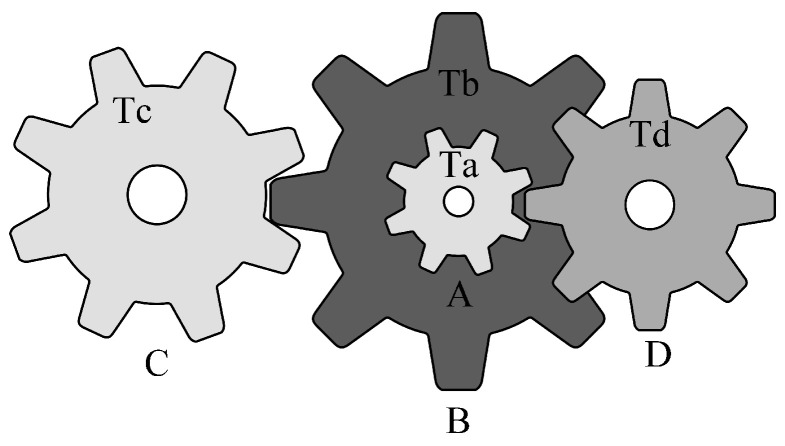
Structure diagram of GTD.

The primary objective of the gear train design problem [45] is to minimize the specific cost of the transmission. As illustrated in Figure 13, the design variables for this problem consist of four gear quantities: $T_{a}$, $T_{b}$, $T_{c}$, and $T_{d}$. The mathematical model is presented in Appendix B.4.

**Figure 14:**
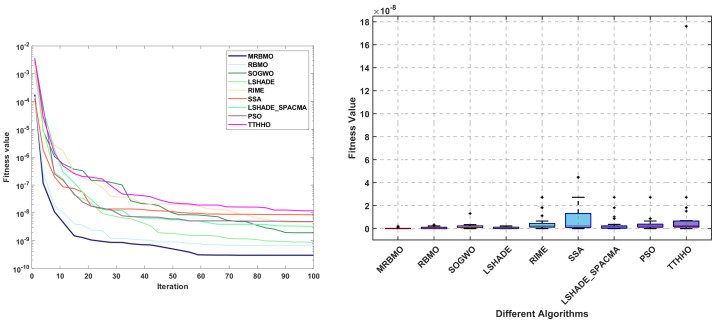
Curve and box plots of GTD.

The statistical results of the algorithms are presented in Table 10. It is evident that all algorithms achieved comparable optimal solutions across the thirty independent trials. In terms of the mean, median, and standard deviation, MRBMO demonstrates enhanced stability in locating superior solutions. Notably, the worst-case values indicate that MRBMO and RBMO are of the same order of magnitude, significantly outperforming other algorithms and further validating their stability in GTD problems. Figure 14 illustrates the convergence curves and box plots, where MRBMO maintains a faster convergence rate throughout the entire process while exhibiting the fewest outliers, which substantiates its superior stability.

## 6. Conclusions

This study proposes a Multi-Strategy Enhanced Red-billed Blue Magpie Optimizer (MRBMO) to address the limitations of convergence efficiency and solution precision inherent in the original RBMO algorithm. The primary innovations of MRBMO include the design of a novel dynamic boundary constraint processing mechanism, the introduction of an elite guidance strategy during the predation stage, and the implementation of a Lévy Flight strategy to adjust the search step size. These synergistic modifications effectively reconcile the exploration–exploitation trade-off in metaheuristic optimization.

Comprehensive experimental evaluations using the standardized CEC2017 and CEC2022 benchmark suites demonstrate that MRBMO outperforms classical optimizers (SOGWO, TTHHO, PSO) and achieves competitive results against a state-of-the-art algorithm (LSHADE_SPACMA). Nonparametric statistical analyses (Friedman rank-sum test and the Wilcoxon signed-rank test) statistically validate MRBMO’s superiority across multiple dimensional configurations (30D, 50D, 100D), confirming its algorithmic robustness. To systematically evaluate the contribution of each methodological enhancement, a comprehensive ablation study was conducted to quantify the individual effects of the proposed algorithmic components on overall performance. Furthermore, an experiment was carried out to assess the method’s capacity for balancing exploration and exploitation mechanisms, while a thorough analysis was performed on the algorithm’s population diversity characteristics to evaluate its evolutionary dynamics.

To validate the performance of the proposed method in practical applications, MRBMO was applied to four engineering design optimization problems: extension/compression spring design, reducer design, welded beam design, and gear train design. The experimental results demonstrate that MRBMO not only achieves superior convergence accuracy and speed but also exhibits enhanced stability and robustness.

## Figures and Tables

**Table 1:** The influence of MRBMO’s parameters (ϵ and β) tested on CEC2022.

Function	Index	Scenario1	Scenario2	Scenario3	Scenario4	Scenario5	Scenario6	Scenario7	Scenario8	Scenario9
Argument	ϵ	0.25	0.25	0.25	0.5	0.5	0.5	0.75	0.75	0.75
	β	0.5	1	1.5	0.5	1	1.5	0.5	1	1.5
F1	Avg	300.40	300.34	300.38	300.23	300.18	300.19	300.12	300.22	300.19
	Rank	9	7	8	6	2	4	1	5	3
F2	Avg	445.55	446.79	444.69	450.30	446.58	451.88	451.47	444.68	442.75
	Rank	4	6	3	7	5	9	8	2	1
F3	Avg	600.11	600.05	600.11	600.07	600.15	600.15	600.04	600.08	600.09
	Rank	6	2	7	3	8	9	1	4	5
F4	Avg	831.93	831.00	827.69	833.18	832.41	832.13	829.29	830.20	830.56
	Rank	6	5	1	9	8	7	2	3	4
F5	Avg	903.62	905.88	902.87	903.31	903.23	902.43	902.19	903.56	901.78
	Rank	8	9	4	6	5	3	2	7	1
F6	Avg	2792.79	2563.47	3744.63	2305.86	2121.38	2210.58	2376.10	2329.08	2172.18
	Rank	8	7	9	4	1	3	6	5	2
F7	Avg	2048.98	2041.51	2048.92	2047.49	2048.30	2047.05	2043.88	2043.35	2041.57
	Rank	9	1	8	6	7	5	4	3	2
F8	Avg	2250.17	2250.40	2238.80	2233.92	2240.76	2229.68	2247.15	2233.63	2237.16
	Rank	8	9	5	3	6	1	7	2	4
F9	Avg	2480.78	2480.78	2480.78	2480.78	2480.78	2480.78	2480.78	2480.78	2480.78
	Rank	3	4	1	7	8	6	2	5	9
F10	Avg	2843.70	2903.50	2660.81	2905.06	2911.99	2848.77	2894.23	2898.44	2937.29
	Rank	2	6	1	7	8	3	4	5	9
F11	Avg	2900.00	2900.00	2900.00	2900.00	2900.00	2900.00	2900.00	2900.00	2900.00
	Rank	5	4	8	2	1	7	3	6	9
F12	Avg	2956.75	2957.06	2963.30	2953.34	2956.41	2952.00	2952.82	2955.63	2956.05
	Rank	7	8	9	3	6	1	2	4	5
Mean Rank		6.3	5.7	5.3	5.3	5.4	4.8	3.5	4.3	4.5

**Table 2:** Ablation experiment of CEC2022.

Function	Index	RBMO	RBMO1	RBMO2	MRBMO
F1	Min	3.0099E+02	3.0004E+02	3.0000E+02	3.0000E+02
	Avg	3.0562E+02	3.0204E+02	3.0030E+02	3.0014E+02
	Std	3.6600E+00	4.4658E+00	4.4298E-01	2.8318E-01
	Rank	4	3	2	1
F2	Min	4.3081E+02	4.0027E+02	4.4490E+02	4.0584E+02
	Avg	4.5500E+02	4.4119E+02	4.5190E+02	4.5086E+02
	Std	1.2485E+01	2.0785E+01	8.2075E+00	1.1773E+01
	Rank	4	1	3	2
F3	Min	6.0001E+02	6.0000E+02	6.0000E+02	6.0000E+02
	Avg	6.0031E+02	6.0066E+02	6.0009E+02	6.0003E+02
	Std	6.0572E-01	1.7808E+00	1.6496E-01	2.4421E-02
	Rank	3	4	2	1
F4	Min	8.1238E+02	8.2190E+02	8.1592E+02	8.1691E+02
	Avg	8.3101E+02	8.3488E+02	8.2683E+02	8.3161E+02
	Std	9.3417E+00	1.0593E+01	9.1196E+00	8.9787E+00
	Rank	2	4	1	3
F5	Min	9.0054E+02	9.0054E+02	9.0000E+02	9.0000E+02
	Avg	9.0578E+02	9.0401E+02	9.0316E+02	9.0330E+02
	Std	5.5217E+00	4.9538E+00	5.7774E+00	3.6224E+00
	Rank	4	3	1	2
F6	Min	1.8573E+03	1.8534E+03	1.8243E+03	1.8465E+03
	Avg	2.4147E+03	3.4106E+03	2.4491E+03	2.1821E+03
	Std	2.2683E+03	3.2690E+03	2.1614E+03	9.7115E+02
	Rank	2	4	3	1
F7	Min	2.0263E+03	2.0260E+03	2.0241E+03	2.0251E+03
	Avg	2.0523E+03	2.0499E+03	2.0428E+03	2.0398E+03
	Std	1.7996E+01	2.2585E+01	1.5680E+01	1.1262E+01
	Rank	4	3	2	1
F8	Min	2.2241E+03	2.2246E+03	2.2214E+03	2.2217E+03
	Avg	2.2462E+03	2.2376E+03	2.2297E+03	2.2300E+03
	Std	4.0668E+01	3.0494E+01	1.8648E+01	2.2493E+01
	Rank	4	3	1	2
F9	Min	2.4808E+03	2.4808E+03	2.4808E+03	2.4808E+03
	Avg	2.4808E+03	2.4808E+03	2.4808E+03	2.4808E+03
	Std	2.0010E-04	1.6344E-08	2.4676E-10	2.6702E-09
	Rank	4	3	1	2
F10	Min	2.5004E+03	2.5003E+03	2.5004E+03	2.5004E+03
	Avg	3.4839E+03	3.0681E+03	3.1715E+03	2.9806E+03
	Std	8.7540E+02	4.9379E+02	6.9863E+02	4.8419E+02
	Rank	4	2	3	1
F11	Min	2.9000E+03	2.9000E+03	2.9000E+03	2.9000E+03
	Avg	2.9001E+03	2.9000E+03	2.9000E+03	2.9000E+03
	Std	4.9301E-02	4.9872E-08	1.3116E-09	6.1406E-10
	Rank	4	3	2	1
F12	Min	2.9397E+03	2.9346E+03	2.9357E+03	2.9328E+03
	Avg	2.9648E+03	2.9786E+03	2.9550E+03	2.9547E+03
	Std	2.3877E+01	5.0664E+01	1.4986E+01	1.5355E+01
	Rank	3	4	2	1
Mean rank		3.50	3.08	1.92	1.50

**Table 3:** Statistics results of 30D.

Function	Index	MRBMO	RBMO	SOGWO	RIME	SSA	LSHADE_SPACMA	PSO	TTHHO
F1	Avg(Rank)	3.85E+03(1)	1.76E+04(3)	5.76E+08(6)	3.81E+06(5)	5.56E+03(2)	9.24E+04(4)	5.78E+08(7)	2.04E+09(8)
	Std(Rank)	4.55E+03(1)	2.65E+04(3)	7.32E+08(6)	1.62E+06(5)	6.98E+03(2)	1.82E+05(4)	9.89E+08(8)	9.46E+08(7)
F2	Avg(Rank)	1.12E+03(1)	1.66E+03(2)	6.09E+04(5)	4.79E+04(4)	4.56E+04(3)	7.51E+04(8)	6.77E+04(7)	6.51E+04(6)
	Std(Rank)	5.35E+02(1)	7.69E+02(2)	1.18E+04(5)	1.46E+04(6)	7.25E+03(4)	6.05E+04(8)	2.62E+04(7)	6.74E+03(3)
F3	Avg(Rank)	4.91E+02(1)	5.13E+02(4)	5.64E+02(6)	5.40E+02(5)	5.01E+02(3)	4.99E+02(2)	6.75E+02(7)	1.04E+03(8)
	Std(Rank)	2.63E+01(2)	2.72E+01(3)	3.45E+01(5)	5.88E+01(6)	3.05E+01(4)	2.36E+01(1)	3.17E+02(8)	2.93E+02(7)
F4	Avg(Rank)	5.62E+02(1)	5.66E+02(2)	6.05E+02(4)	6.07E+02(5)	7.30E+02(7)	5.80E+02(3)	6.21E+02(6)	7.80E+02(8)
	Std(Rank)	1.88E+01(2)	1.62E+01(1)	3.33E+01(6)	2.58E+01(4)	4.66E+01(8)	1.95E+01(3)	3.64E+01(7)	3.22E+01(5)
F5	Avg(Rank)	6.01E+02(1)	6.02E+02(2)	6.08E+02(4)	6.15E+02(6)	6.48E+02(7)	6.02E+02(3)	6.12E+02(5)	6.70E+02(8)
	Std(Rank)	4.61E-01(1)	2.08E+00(3)	3.62E+00(4)	5.60E+00(5)	1.09E+01(8)	1.23E+00(2)	6.21E+00(7)	6.21E+00(6)
F6	Avg(Rank)	7.90E+02(1)	8.02E+02(2)	8.79E+02(5)	8.78E+02(4)	1.23E+03(7)	8.32E+02(3)	8.91E+02(6)	1.32E+03(8)
	Std(Rank)	1.63E+01(1)	2.21E+01(2)	5.62E+01(6)	3.52E+01(4)	1.03E+02(8)	3.05E+01(3)	4.66E+01(5)	6.62E+01(7)
F7	Avg(Rank)	8.59E+02(1)	8.70E+02(2)	9.05E+02(4)	9.18E+02(6)	9.73E+02(7)	8.78E+02(3)	9.12E+02(5)	9.94E+02(8)
	Std(Rank)	1.58E+01(1)	2.15E+01(3)	4.63E+01(8)	2.41E+01(5)	3.62E+01(7)	1.99E+01(2)	2.65E+01(6)	2.38E+01(4)
F8	Avg(Rank)	9.32E+02(1)	1.01E+03(2)	2.20E+03(4)	3.23E+03(6)	5.26E+03(7)	1.23E+03(3)	2.71E+03(5)	8.60E+03(8)
	Std(Rank)	3.56E+01(1)	1.17E+02(2)	1.47E+03(6)	1.81E+03(7)	4.22E+02(4)	2.61E+02(3)	1.81E+03(8)	1.34E+03(5)
F9	Avg(Rank)	4.25E+03(1)	4.91E+03(5)	5.00E+03(6)	4.79E+03(3)	5.39E+03(7)	4.85E+03(4)	4.65E+03(2)	7.29E+03(8)
	Std(Rank)	5.95E+02(3)	5.61E+02(2)	1.15E+03(8)	6.95E+02(4)	5.35E+02(1)	8.18E+02(6)	7.00E+02(5)	9.63E+02(7)
F10	Avg(Rank)	1.18E+03(1)	1.22E+03(2)	1.89E+03(7)	1.34E+03(6)	1.32E+03(5)	1.26E+03(3)	1.28E+03(4)	2.26E+03(8)
	Std(Rank)	3.73E+01(1)	3.76E+01(2)	7.98E+02(8)	6.46E+01(6)	6.09E+01(5)	5.57E+01(4)	5.38E+01(3)	3.71E+02(7)
F11	Avg(Rank)	6.83E+04(1)	9.78E+04(2)	7.01E+07(6)	1.85E+07(5)	1.51E+06(4)	3.66E+05(3)	1.04E+08(7)	2.42E+08(8)
	Std(Rank)	6.41E+04(1)	9.22E+04(2)	9.97E+07(6)	2.12E+07(5)	1.19E+06(4)	4.02E+05(3)	2.77E+08(8)	2.14E+08(7)
F12	Avg(Rank)	1.11E+04(1)	1.84E+04(3)	7.00E+06(7)	2.71E+05(5)	3.36E+04(4)	1.44E+04(2)	7.35E+06(8)	2.07E+06(6)
	Std(Rank)	1.34E+04(2)	2.05E+04(3)	1.52E+07(7)	4.25E+05(5)	2.50E+04(4)	9.27E+03(1)	2.18E+07(8)	1.16E+06(6)
F13	Avg(Rank)	1.48E+03(1)	1.51E+03(2)	5.36E+05(7)	1.07E+05(6)	5.42E+04(4)	1.09E+04(3)	5.46E+04(5)	1.09E+06(8)
	Std(Rank)	1.64E+01(1)	2.12E+01(2)	8.33E+05(8)	8.33E+04(6)	5.24E+04(5)	5.04E+04(3)	5.23E+04(4)	6.94E+05(7)
F14	Avg(Rank)	1.88E+03(1)	2.23E+03(2)	2.23E+05(8)	1.72E+04(6)	9.09E+03(4)	2.55E+03(3)	1.02E+04(5)	1.86E+05(7)
	Std(Rank)	3.06E+02(1)	5.09E+02(2)	5.09E+05(8)	1.05E+04(5)	8.67E+03(4)	6.67E+02(3)	1.17E+04(6)	1.02E+05(7)
F15	Avg(Rank)	2.26E+03(1)	2.48E+03(2)	2.69E+03(5)	2.86E+03(6)	2.91E+03(7)	2.63E+03(3)	2.64E+03(4)	3.95E+03(8)
	Std(Rank)	2.45E+02(1)	3.34E+02(5)	3.58E+02(7)	2.67E+02(2)	3.11E+02(4)	2.76E+02(3)	3.57E+02(6)	5.56E+02(8)
F16	Avg(Rank)	1.90E+03(1)	2.04E+03(3)	2.07E+03(4)	2.20E+03(6)	2.50E+03(7)	1.99E+03(2)	2.17E+03(5)	2.78E+03(8)
	Std(Rank)	9.31E+01(1)	1.53E+02(3)	1.73E+02(4)	2.31E+02(6)	2.77E+02(7)	1.46E+02(2)	1.88E+02(5)	2.82E+02(8)
F17	Avg(Rank)	2.56E+03(2)	2.42E+03(1)	1.67E+06(6)	1.30E+06(5)	5.13E+05(4)	3.64E+04(3)	1.69E+06(7)	1.17E+07(8)
	Std(Rank)	1.27E+03(2)	3.93E+02(1)	2.01E+06(7)	1.51E+06(5)	7.25E+05(4)	1.96E+04(3)	1.94E+06(6)	1.59E+07(8)
F18	Avg(Rank)	2.00E+03(1)	4.23E+03(3)	2.12E+06(7)	1.76E+04(5)	9.91E+03(4)	2.14E+03(2)	9.27E+04(6)	3.79E+06(8)
	Std(Rank)	5.85E+01(1)	8.74E+03(3)	7.87E+06(8)	1.30E+04(4)	1.37E+04(5)	1.46E+02(2)	2.55E+05(6)	2.98E+06(7)
F19	Avg(Rank)	2.29E+03(1)	2.38E+03(2)	2.48E+03(4)	2.51E+03(6)	2.77E+03(7)	2.50E+03(5)	2.44E+03(3)	2.90E+03(8)
	Std(Rank)	1.42E+02(3)	1.27E+02(2)	1.46E+02(4)	1.94E+02(5)	2.31E+02(7)	1.02E+02(1)	2.42E+02(8)	2.19E+02(6)
F20	Avg(Rank)	2.36E+03(1)	2.37E+03(2)	2.40E+03(4)	2.43E+03(6)	2.52E+03(7)	2.38E+03(3)	2.41E+03(5)	2.60E+03(8)
	Std(Rank)	2.10E+01(2)	1.88E+01(1)	3.03E+01(5)	3.19E+01(6)	5.15E+01(7)	2.80E+01(4)	2.66E+01(3)	7.38E+01(8)
F21	Avg(Rank)	2.55E+03(2)	4.45E+03(3)	5.65E+03(6)	5.03E+03(5)	6.47E+03(7)	2.31E+03(1)	4.64E+03(4)	7.93E+03(8)
	Std(Rank)	9.30E+02(2)	2.11E+03(7)	2.19E+03(8)	1.86E+03(5)	1.53E+03(4)	2.01E+01(1)	1.90E+03(6)	1.31E+03(3)
F22	Avg(Rank)	2.72E+03(1)	2.75E+03(3)	2.76E+03(4)	2.78E+03(5)	2.93E+03(7)	2.73E+03(2)	2.90E+03(6)	3.30E+03(8)
	Std(Rank)	2.67E+01(2)	3.01E+01(4)	3.10E+01(5)	2.82E+01(3)	7.70E+01(6)	2.11E+01(1)	8.99E+01(7)	1.03E+02(8)
F23	Avg(Rank)	2.88E+03(1)	2.93E+03(3)	2.95E+03(5)	2.94E+03(4)	3.10E+03(6)	2.91E+03(2)	3.10E+03(7)	3.55E+03(8)
	Std(Rank)	2.00E+01(1)	2.94E+01(3)	6.73E+01(5)	3.85E+01(4)	8.52E+01(7)	2.57E+01(2)	8.25E+01(6)	1.73E+02(8)
F24	Avg(Rank)	2.89E+03(1)	2.89E+03(2)	2.96E+03(7)	2.92E+03(6)	2.89E+03(3)	2.90E+03(4)	2.91E+03(5)	3.06E+03(8)
	Std(Rank)	1.40E+01(3)	6.10E+00(1)	4.34E+01(6)	2.99E+01(5)	1.30E+01(2)	1.51E+01(4)	4.69E+01(8)	4.45E+01(7)
F25	Avg(Rank)	4.22E+03(1)	4.75E+03(3)	4.87E+03(4)	5.09E+03(6)	6.05E+03(7)	4.37E+03(2)	4.97E+03(5)	8.18E+03(8)
	Std(Rank)	5.83E+02(2)	8.01E+02(5)	4.02E+02(1)	6.23E+02(4)	1.28E+03(8)	5.91E+02(3)	9.83E+02(6)	1.25E+03(7)
F26	Avg(Rank)	3.22E+03(1)	3.23E+03(2)	3.26E+03(5)	3.24E+03(4)	3.27E+03(6)	3.24E+03(3)	3.29E+03(7)	3.70E+03(8)
	Std(Rank)	1.43E+01(1)	2.29E+01(5)	1.71E+01(4)	1.63E+01(3)	3.91E+01(6)	1.45E+01(2)	4.62E+01(7)	2.21E+02(8)
F27	Avg(Rank)	3.21E+03(1)	3.28E+03(4)	3.38E+03(7)	3.28E+03(5)	3.23E+03(2)	3.25E+03(3)	3.33E+03(6)	3.65E+03(8)
	Std(Rank)	2.30E+01(2)	4.63E+01(5)	6.97E+01(6)	3.41E+01(3)	2.19E+01(1)	3.65E+01(4)	1.19E+02(7)	1.51E+02(8)
F28	Avg(Rank)	3.63E+03(2)	3.82E+03(3)	3.86E+03(5)	4.04E+03(6)	4.26E+03(7)	3.62E+03(1)	3.85E+03(4)	5.45E+03(8)
	Std(Rank)	1.43E+02(3)	1.33E+02(2)	1.54E+02(4)	2.35E+02(6)	2.53E+02(7)	1.06E+02(1)	2.22E+02(5)	7.66E+02(8)
F29	Avg(Rank)	1.44E+04(1)	5.21E+04(4)	1.11E+07(7)	6.76E+05(6)	1.88E+04(2)	1.97E+04(3)	2.60E+05(5)	8.09E+07(8)
	Std(Rank)	7.76E+03(1)	1.31E+05(4)	9.27E+06(7)	5.52E+05(5)	1.21E+04(3)	8.23E+03(2)	7.43E+05(6)	1.29E+08(8)

**Table 4:** Statistics results of 50D.

Function	Index	MRBMO	RBMO	SOGWO	RIME	SSA	LSHADE_SPACMA	PSO	TTHHO
F1	Avg(Rank)	6.40E+03(1)	4.74E+07(4)	6.52E+09(7)	4.33E+07(3)	9.66E+05(2)	9.62E+07(5)	4.25E+09(6)	1.14E+10(8)
	Std(Rank)	6.58E+03(1)	7.26E+07(4)	3.89E+09(7)	1.75E+07(3)	5.66E+05(2)	1.01E+08(5)	5.00E+09(8)	3.06E+09(6)
F2	Avg(Rank)	3.13E+04(1)	3.74E+04(2)	1.76E+05(4)	2.09E+05(7)	2.59E+05(8)	1.42E+05(3)	1.98E+05(6)	1.88E+05(5)
	Std(Rank)	1.36E+04(1)	1.62E+04(2)	2.72E+04(4)	4.76E+04(5)	7.33E+04(7)	8.05E+04(8)	4.87E+04(6)	2.04E+04(3)
F3	Avg(Rank)	5.49E+02(1)	6.41E+02(3)	1.08E+03(7)	6.99E+02(5)	5.70E+02(2)	6.64E+02(4)	9.63E+02(6)	3.07E+03(8)
	Std(Rank)	3.80E+01(1)	6.13E+01(4)	3.18E+02(6)	6.55E+01(5)	4.84E+01(3)	4.74E+01(2)	4.98E+02(7)	8.03E+02(8)
F4	Avg(Rank)	6.37E+02(1)	6.95E+02(2)	7.76E+02(5)	7.55E+02(4)	8.66E+02(7)	7.22E+02(3)	7.77E+02(6)	9.66E+02(8)
	Std(Rank)	2.68E+01(1)	3.85E+01(5)	9.17E+01(8)	4.72E+01(6)	3.02E+01(2)	3.82E+01(4)	5.18E+01(7)	3.57E+01(3)
F5	Avg(Rank)	6.05E+02(1)	6.08E+02(2)	6.20E+02(4)	6.32E+02(5)	6.63E+02(7)	6.09E+02(3)	6.34E+02(6)	6.82E+02(8)
	Std(Rank)	2.09E+00(1)	3.13E+00(3)	5.31E+00(4)	8.16E+00(7)	5.66E+00(5)	2.97E+00(2)	1.28E+01(8)	5.92E+00(6)
F6	Avg(Rank)	9.11E+02(1)	9.92E+02(2)	1.13E+03(5)	1.12E+03(4)	1.73E+03(7)	1.09E+03(3)	1.18E+03(6)	1.91E+03(8)
	Std(Rank)	2.91E+01(1)	5.31E+01(3)	9.74E+01(8)	7.39E+01(5)	5.17E+01(2)	7.31E+01(4)	7.73E+01(7)	7.72E+01(6)
F7	Avg(Rank)	9.46E+02(1)	1.01E+03(2)	1.05E+03(4)	1.06E+03(5)	1.18E+03(7)	1.01E+03(3)	1.09E+03(6)	1.26E+03(8)
	Std(Rank)	2.77E+01(1)	4.47E+01(5)	6.11E+01(6)	6.58E+01(8)	4.16E+01(3)	4.41E+01(4)	6.19E+01(7)	3.34E+01(2)
F8	Avg(Rank)	1.66E+03(1)	2.52E+03(2)	9.40E+03(4)	1.24E+04(5)	1.32E+04(6)	4.99E+03(3)	2.07E+04(7)	3.38E+04(8)
	Std(Rank)	5.54E+02(1)	8.07E+02(3)	4.56E+03(6)	6.91E+03(8)	7.89E+02(2)	2.40E+03(4)	6.69E+03(7)	2.91E+03(5)
F9	Avg(Rank)	7.57E+03(1)	9.51E+03(7)	8.69E+03(5)	8.06E+03(3)	8.37E+03(4)	9.14E+03(6)	8.03E+03(2)	1.08E+04(8)
	Std(Rank)	8.42E+02(2)	1.19E+03(6)	2.74E+03(8)	7.21E+02(1)	9.07E+02(3)	1.37E+03(7)	1.15E+03(5)	1.05E+03(4)
F10	Avg(Rank)	1.28E+03(1)	1.44E+03(3)	5.27E+03(8)	1.72E+03(4)	1.41E+03(2)	2.71E+03(6)	1.72E+03(5)	4.58E+03(7)
	Std(Rank)	4.30E+01(1)	8.29E+01(3)	1.86E+03(7)	1.13E+02(4)	7.12E+01(2)	3.48E+03(8)	1.99E+02(5)	8.86E+02(6)
F11	Avg(Rank)	2.31E+06(1)	1.11E+07(2)	6.77E+08(6)	1.62E+08(5)	1.32E+07(3)	1.36E+07(4)	2.91E+09(7)	3.14E+09(8)
	Std(Rank)	1.82E+06(1)	8.74E+06(3)	9.61E+08(6)	1.01E+08(5)	8.51E+06(2)	1.26E+07(4)	2.72E+09(8)	2.02E+09(7)
F12	Avg(Rank)	7.71E+03(1)	2.13E+04(3)	9.67E+07(6)	4.46E+05(5)	3.50E+04(4)	2.06E+04(2)	4.03E+08(8)	3.15E+08(7)
	Std(Rank)	8.52E+03(2)	1.47E+04(3)	1.06E+08(6)	3.54E+05(5)	1.98E+04(4)	8.36E+03(1)	9.36E+08(8)	5.22E+08(7)
F13	Avg(Rank)	1.68E+03(1)	1.77E+03(2)	1.58E+06(7)	6.25E+05(5)	3.96E+05(4)	1.67E+04(3)	7.05E+05(6)	1.45E+07(8)
	Std(Rank)	7.83E+01(1)	8.24E+01(2)	1.15E+06(7)	4.23E+05(5)	2.41E+05(4)	2.25E+04(3)	6.54E+05(6)	1.70E+07(8)
F14	Avg(Rank)	7.94E+03(1)	1.14E+04(3)	1.06E+07(7)	1.12E+05(6)	1.95E+04(5)	1.00E+04(2)	1.39E+04(4)	2.81E+07(8)
	Std(Rank)	5.07E+03(1)	6.64E+03(3)	1.86E+07(7)	5.30E+04(6)	1.11E+04(4)	5.83E+03(2)	1.25E+04(5)	6.15E+07(8)
F15	Avg(Rank)	3.01E+03(1)	3.41E+03(4)	3.22E+03(2)	3.68E+03(6)	3.92E+03(7)	3.45E+03(5)	3.30E+03(3)	5.72E+03(8)
	Std(Rank)	4.14E+02(2)	4.80E+02(6)	6.22E+02(7)	4.40E+02(3)	4.60E+02(5)	3.33E+02(1)	4.54E+02(4)	7.15E+02(8)
F16	Avg(Rank)	2.86E+03(1)	3.14E+03(3)	3.02E+03(2)	3.49E+03(6)	3.58E+03(7)	3.15E+03(4)	3.17E+03(5)	3.85E+03(8)
	Std(Rank)	2.81E+02(1)	3.27E+02(2)	3.91E+02(5)	4.01E+02(6)	4.33E+02(7)	3.34E+02(3)	4.40E+02(8)	3.79E+02(4)
F17	Avg(Rank)	9.35E+04(2)	6.19E+04(1)	5.63E+06(7)	4.70E+06(6)	2.95E+06(4)	5.24E+05(3)	3.34E+06(5)	2.14E+07(8)
	Std(Rank)	1.32E+05(2)	3.54E+04(1)	5.16E+06(7)	3.10E+06(5)	1.76E+06(4)	1.49E+06(3)	3.48E+06(6)	1.66E+07(8)
F18	Avg(Rank)	1.48E+04(2)	1.27E+04(1)	3.17E+06(8)	5.63E+05(5)	1.85E+04(4)	1.50E+04(3)	7.17E+05(6)	2.98E+06(7)
	Std(Rank)	1.10E+04(2)	1.13E+04(3)	3.59E+06(8)	6.40E+05(5)	1.49E+04(4)	9.79E+03(1)	2.44E+06(6)	2.59E+06(7)
F19	Avg(Rank)	2.91E+03(1)	3.12E+03(3)	3.10E+03(2)	3.22E+03(5)	3.60E+03(7)	3.54E+03(6)	3.18E+03(4)	3.60E+03(8)
	Std(Rank)	2.12E+02(1)	2.89E+02(3)	4.04E+02(8)	2.96E+02(4)	3.01E+02(5)	2.41E+02(2)	3.23E+02(6)	3.59E+02(7)
F20	Avg(Rank)	2.45E+03(1)	2.50E+03(2)	2.56E+03(4)	2.57E+03(5)	2.73E+03(7)	2.52E+03(3)	2.60E+03(6)	2.99E+03(8)
	Std(Rank)	3.17E+01(1)	4.25E+01(3)	8.97E+01(6)	5.15E+01(4)	1.06E+02(8)	3.77E+01(2)	6.62E+01(5)	9.27E+01(7)
F21	Avg(Rank)	8.89E+03(1)	1.13E+04(7)	1.03E+04(5)	9.88E+03(2)	1.02E+04(4)	1.13E+04(6)	9.95E+03(3)	1.31E+04(8)
	Std(Rank)	2.01E+03(7)	9.95E+02(3)	1.48E+03(6)	9.95E+02(2)	9.24E+02(1)	2.13E+03(8)	1.09E+03(4)	1.22E+03(5)
F22	Avg(Rank)	2.89E+03(1)	3.06E+03(5)	3.00E+03(3)	3.06E+03(4)	3.35E+03(7)	3.00E+03(2)	3.33E+03(6)	4.10E+03(8)
	Std(Rank)	3.65E+01(1)	8.41E+01(4)	4.44E+01(2)	9.00E+01(5)	1.74E+02(7)	5.76E+01(3)	1.48E+02(6)	2.27E+02(8)
F23	Avg(Rank)	3.05E+03(1)	3.22E+03(5)	3.21E+03(4)	3.19E+03(3)	3.49E+03(6)	3.16E+03(2)	3.54E+03(7)	4.52E+03(8)
	Std(Rank)	4.40E+01(1)	8.58E+01(4)	1.16E+02(5)	6.53E+01(3)	1.53E+02(6)	5.66E+01(2)	1.68E+02(7)	2.38E+02(8)
F24	Avg(Rank)	3.07E+03(1)	3.16E+03(5)	3.60E+03(7)	3.14E+03(3)	3.10E+03(2)	3.15E+03(4)	3.30E+03(6)	4.09E+03(8)
	Std(Rank)	3.11E+01(1)	4.90E+01(5)	4.71E+02(7)	4.89E+01(4)	3.14E+01(2)	3.91E+01(3)	4.79E+02(8)	2.80E+02(6)
F25	Avg(Rank)	5.10E+03(1)	6.28E+03(2)	6.76E+03(5)	7.26E+03(6)	8.10E+03(7)	6.48E+03(3)	6.71E+03(4)	1.21E+04(8)
	Std(Rank)	9.68E+02(5)	1.13E+03(6)	5.56E+02(1)	6.18E+02(3)	3.50E+03(8)	5.57E+02(2)	1.82E+03(7)	8.37E+02(4)
F26	Avg(Rank)	3.35E+03(1)	3.50E+03(2)	3.60E+03(4)	3.60E+03(5)	3.69E+03(6)	3.54E+03(3)	3.77E+03(7)	5.03E+03(8)
	Std(Rank)	6.69E+01(1)	1.21E+02(5)	8.60E+01(2)	1.10E+02(4)	2.33E+02(6)	1.05E+02(3)	2.50E+02(7)	5.80E+02(8)
F27	Avg(Rank)	3.34E+03(1)	3.78E+03(5)	4.25E+03(7)	3.45E+03(3)	3.38E+03(2)	3.48E+03(4)	4.01E+03(6)	5.51E+03(8)
	Std(Rank)	3.10E+01(1)	6.37E+02(7)	3.70E+02(5)	5.65E+01(3)	3.25E+01(2)	8.69E+01(4)	9.33E+02(8)	4.94E+02(6)
F28	Avg(Rank)	4.02E+03(1)	4.63E+03(3)	4.80E+03(4)	5.35E+03(7)	5.12E+03(6)	4.24E+03(2)	4.82E+03(5)	8.66E+03(8)
	Std(Rank)	3.05E+02(2)	4.75E+02(7)	3.51E+02(3)	4.44E+02(6)	3.89E+02(5)	2.98E+02(1)	3.84E+02(4)	1.30E+03(8)
F29	Avg(Rank)	1.25E+06(1)	3.30E+06(3)	1.38E+08(7)	6.30E+07(6)	1.78E+06(2)	3.46E+06(4)	4.09E+06(5)	1.71E+08(8)
	Std(Rank)	3.84E+05(1)	1.70E+06(4)	3.95E+07(7)	2.16E+07(6)	8.37E+05(2)	1.55E+06(3)	3.12E+06(5)	7.36E+07(8)

**Table 5:** Statistics results of 100D.

Function	Index	MRBMO	RBMO	SOGWO	RIME	SSA	LSHADE_SPACMA	PSO	TTHHO
F1	Avg(Rank)	5.56E+07(1)	6.49E+09(4)	4.37E+10(7)	9.59E+08(3)	3.62E+08(2)	8.35E+09(5)	1.92E+10(6)	7.06E+10(8)
	Std(Rank)	3.31E+07(1)	2.15E+09(4)	8.69E+09(7)	2.23E+08(3)	1.41E+08(2)	3.34E+09(5)	1.03E+10(8)	7.24E+09(6)
F2	Avg(Rank)	2.42E+05(2)	2.32E+05(1)	5.56E+05(5)	7.04E+05(7)	7.69E+05(8)	4.53E+05(4)	5.70E+05(6)	3.98E+05(3)
	Std(Rank)	3.56E+04(2)	3.32E+04(1)	6.98E+04(3)	9.83E+04(4)	1.37E+05(6)	1.57E+05(8)	1.14E+05(5)	1.53E+05(7)
F3	Avg(Rank)	8.56E+02(1)	1.73E+03(4)	4.62E+03(7)	1.15E+03(3)	1.02E+03(2)	1.80E+03(5)	3.29E+03(6)	1.46E+04(8)
	Std(Rank)	7.22E+01(1)	3.59E+02(5)	1.55E+03(6)	1.32E+02(3)	7.48E+01(2)	3.54E+02(4)	1.73E+03(7)	2.79E+03(8)
F4	Avg(Rank)	9.81E+02(1)	1.16E+03(2)	1.21E+03(3)	1.32E+03(5)	1.37E+03(6)	1.27E+03(4)	1.47E+03(7)	1.72E+03(8)
	Std(Rank)	8.12E+01(4)	8.87E+01(5)	6.25E+01(3)	1.15E+02(8)	4.10E+01(1)	9.56E+01(6)	1.14E+02(7)	5.67E+01(2)
F5	Avg(Rank)	6.23E+02(1)	6.32E+02(3)	6.45E+02(4)	6.56E+02(5)	6.66E+02(7)	6.28E+02(2)	6.64E+02(6)	6.94E+02(8)
	Std(Rank)	5.64E+00(5)	5.92E+00(6)	5.23E+00(4)	8.77E+00(7)	2.70E+00(1)	4.40E+00(2)	9.65E+00(8)	4.54E+00(3)
F6	Avg(Rank)	1.51E+03(1)	1.86E+03(2)	2.17E+03(3)	2.21E+03(4)	3.26E+03(7)	2.26E+03(6)	2.22E+03(5)	3.81E+03(8)
	Std(Rank)	1.36E+02(2)	1.53E+02(4)	1.62E+02(5)	2.14E+02(8)	7.33E+01(1)	2.05E+02(7)	1.79E+02(6)	1.41E+02(3)
F7	Avg(Rank)	1.27E+03(1)	1.47E+03(2)	1.58E+03(3)	1.63E+03(5)	1.83E+03(7)	1.61E+03(4)	1.77E+03(6)	2.19E+03(8)
	Std(Rank)	6.13E+01(2)	8.37E+01(4)	1.66E+02(8)	1.36E+02(7)	5.02E+01(1)	1.07E+02(5)	1.24E+02(6)	6.42E+01(3)
F8	Avg(Rank)	1.22E+04(1)	1.94E+04(2)	4.59E+04(5)	4.92E+04(6)	2.52E+04(3)	3.37E+04(4)	7.03E+04(7)	7.25E+04(8)
	Std(Rank)	3.92E+03(2)	4.76E+03(3)	1.25E+04(7)	1.20E+04(6)	6.32E+02(1)	1.03E+04(5)	1.76E+04(8)	5.14E+03(4)
F9	Avg(Rank)	1.93E+04(2)	2.34E+04(6)	2.05E+04(4)	2.00E+04(3)	1.75E+04(1)	2.44E+04(7)	2.11E+04(5)	2.63E+04(8)
	Std(Rank)	1.14E+03(1)	1.80E+03(4)	5.51E+03(8)	1.31E+03(2)	1.41E+03(3)	3.41E+03(7)	2.86E+03(6)	2.63E+03(5)
F10	Avg(Rank)	5.44E+03(1)	1.23E+04(2)	8.62E+04(7)	4.47E+04(3)	7.80E+04(6)	6.11E+04(4)	7.58E+04(5)	1.98E+05(8)
	Std(Rank)	1.58E+03(1)	3.61E+03(2)	1.97E+04(4)	1.14E+04(3)	2.29E+04(5)	3.83E+04(7)	2.88E+04(6)	5.42E+04(8)
F11	Avg(Rank)	2.90E+07(1)	3.58E+08(3)	9.37E+09(7)	1.25E+09(5)	1.76E+08(2)	9.27E+08(4)	8.90E+09(6)	2.22E+10(8)
	Std(Rank)	1.33E+07(1)	2.01E+08(3)	4.60E+09(6)	4.58E+08(5)	8.34E+07(2)	4.13E+08(4)	7.29E+09(8)	6.26E+09(7)
F12	Avg(Rank)	8.46E+03(1)	6.90E+05(3)	1.25E+09(7)	2.18E+06(5)	1.25E+05(2)	1.53E+06(4)	9.82E+08(6)	1.56E+09(8)
	Std(Rank)	5.26E+03(1)	3.46E+06(4)	8.14E+08(6)	1.42E+06(3)	3.41E+05(2)	4.83E+06(5)	1.30E+09(8)	8.36E+08(7)
F13	Avg(Rank)	3.21E+05(2)	3.07E+05(1)	7.19E+06(6)	8.00E+06(7)	1.99E+06(4)	8.82E+05(3)	5.61E+06(5)	1.53E+07(8)
	Std(Rank)	3.80E+05(2)	2.97E+05(1)	3.99E+06(6)	4.15E+06(7)	9.09E+05(4)	5.02E+05(3)	2.87E+06(5)	5.29E+06(8)
F14	Avg(Rank)	5.76E+03(1)	4.42E+05(5)	2.17E+08(7)	4.24E+05(4)	2.72E+04(2)	3.03E+04(3)	2.22E+08(8)	1.06E+08(6)
	Std(Rank)	5.28E+03(1)	1.99E+06(5)	2.67E+08(7)	2.41E+05(4)	1.66E+04(2)	1.78E+04(3)	4.63E+08(8)	8.09E+07(6)
F15	Avg(Rank)	5.49E+03(1)	6.72E+03(5)	6.59E+03(4)	7.33E+03(7)	6.11E+03(2)	7.08E+03(6)	6.36E+03(3)	1.21E+04(8)
	Std(Rank)	6.05E+02(2)	9.36E+02(6)	8.14E+02(4)	6.62E+02(3)	5.17E+02(1)	9.56E+02(7)	8.90E+02(5)	1.79E+03(8)
F16	Avg(Rank)	4.72E+03(1)	5.50E+03(3)	5.45E+03(2)	5.91E+03(5)	5.99E+03(6)	5.79E+03(4)	6.48E+03(7)	1.12E+04(8)
	Std(Rank)	5.06E+02(2)	4.64E+02(1)	1.89E+03(7)	6.07E+02(3)	6.16E+02(4)	8.31E+02(5)	1.84E+03(6)	3.47E+03(8)
F17	Avg(Rank)	1.06E+06(2)	6.20E+05(1)	7.97E+06(5)	1.12E+07(7)	3.02E+06(4)	1.45E+06(3)	8.78E+06(6)	1.30E+07(8)
	Std(Rank)	6.77E+05(2)	3.52E+05(1)	4.49E+06(6)	5.26E+06(7)	1.83E+06(4)	6.89E+05(3)	4.05E+06(5)	7.64E+06(8)
F18	Avg(Rank)	6.31E+03(1)	1.36E+04(2)	4.04E+08(8)	2.62E+07(5)	1.98E+04(3)	1.26E+05(4)	2.76E+08(7)	1.39E+08(6)
	Std(Rank)	4.65E+03(1)	1.65E+04(2)	5.69E+08(7)	1.60E+07(5)	2.52E+04(3)	1.22E+05(4)	7.06E+08(8)	1.63E+08(6)
F19	Avg(Rank)	4.89E+03(1)	5.23E+03(2)	5.60E+03(4)	5.57E+03(3)	6.08E+03(6)	6.63E+03(8)	5.65E+03(5)	6.58E+03(7)
	Std(Rank)	5.40E+02(3)	5.64E+02(4)	1.16E+03(8)	6.12E+02(5)	6.77E+02(6)	4.33E+02(1)	6.84E+02(7)	5.35E+02(2)
F20	Avg(Rank)	2.80E+03(1)	3.11E+03(3)	3.09E+03(2)	3.22E+03(5)	3.68E+03(7)	3.13E+03(4)	3.50E+03(6)	4.50E+03(8)
	Std(Rank)	7.40E+01(2)	1.02E+02(4)	8.59E+01(3)	1.30E+02(5)	2.73E+02(8)	6.77E+01(1)	1.42E+02(6)	1.93E+02(7)
F21	Avg(Rank)	2.12E+04(2)	2.46E+04(6)	2.27E+04(4)	2.23E+04(3)	2.00E+04(1)	2.62E+04(7)	2.37E+04(5)	2.91E+04(8)
	Std(Rank)	1.56E+03(4)	1.96E+03(5)	5.15E+03(8)	1.48E+03(2)	1.39E+03(1)	2.62E+03(7)	2.58E+03(6)	1.49E+03(3)
F22	Avg(Rank)	3.36E+03(1)	3.91E+03(5)	3.65E+03(3)	3.74E+03(4)	4.32E+03(6)	3.63E+03(2)	4.73E+03(7)	6.26E+03(8)
	Std(Rank)	1.15E+02(4)	1.70E+02(5)	1.09E+02(3)	1.00E+02(2)	1.88E+02(6)	9.11E+01(1)	2.82E+02(7)	5.63E+02(8)
F23	Avg(Rank)	3.84E+03(1)	4.64E+03(5)	4.37E+03(3)	4.24E+03(2)	5.25E+03(6)	4.43E+03(4)	5.73E+03(7)	9.30E+03(8)
	Std(Rank)	9.54E+01(1)	2.05E+02(5)	1.04E+02(2)	1.57E+02(4)	4.60E+02(7)	1.55E+02(3)	4.50E+02(6)	1.20E+03(8)
F24	Avg(Rank)	3.55E+03(1)	4.39E+03(4)	6.91E+03(7)	3.91E+03(3)	3.70E+03(2)	4.41E+03(5)	4.59E+03(6)	8.47E+03(8)
	Std(Rank)	6.57E+01(1)	2.47E+02(4)	9.75E+02(8)	1.33E+02(3)	7.66E+01(2)	3.13E+02(5)	7.21E+02(7)	7.14E+02(6)
F25	Avg(Rank)	1.15E+04(1)	1.58E+04(3)	1.69E+04(4)	1.57E+04(2)	2.30E+04(7)	1.75E+04(5)	1.95E+04(6)	3.41E+04(8)
	Std(Rank)	2.42E+03(4)	2.60E+03(5)	1.27E+03(1)	1.45E+03(2)	6.53E+03(8)	1.82E+03(3)	2.92E+03(7)	2.65E+03(6)
F26	Avg(Rank)	3.60E+03(1)	3.72E+03(2)	4.19E+03(6)	4.09E+03(5)	3.81E+03(3)	4.04E+03(4)	4.20E+03(7)	8.18E+03(8)
	Std(Rank)	7.68E+01(1)	1.18E+02(2)	1.74E+02(4)	1.57E+02(3)	1.87E+02(5)	1.99E+02(6)	3.93E+02(7)	1.73E+03(8)
F27	Avg(Rank)	3.67E+03(1)	6.67E+03(5)	8.80E+03(7)	4.31E+03(3)	3.83E+03(2)	5.48E+03(4)	6.89E+03(6)	1.15E+04(8)
	Std(Rank)	7.66E+01(1)	2.37E+03(8)	1.40E+03(6)	4.07E+02(3)	8.36E+01(2)	7.14E+02(4)	1.74E+03(7)	8.72E+02(5)
F28	Avg(Rank)	7.03E+03(1)	8.08E+03(5)	8.95E+03(6)	9.66E+03(7)	7.77E+03(2)	7.99E+03(3)	8.01E+03(4)	1.45E+04(8)
	Std(Rank)	5.90E+02(1)	6.80E+02(5)	7.40E+02(7)	7.36E+02(6)	5.90E+02(2)	6.31E+02(4)	5.95E+02(3)	1.96E+03(8)
F29	Avg(Rank)	2.60E+04(1)	1.08E+06(3)	1.33E+09(7)	1.79E+08(5)	8.09E+05(2)	9.38E+06(4)	8.44E+08(6)	1.83E+09(8)
	Std(Rank)	1.22E+04(1)	9.20E+05(3)	1.09E+09(8)	8.03E+07(5)	4.23E+05(2)	6.80E+06(4)	9.14E+08(6)	1.01E+09(7)

**Table 6:** Overall results of the Wilcoxon rank sum test on CEC2017.

Index	R	RBMO	SOGWO	RIME	SSA	LSHADE_SPACMA	PSO	TTHHO
30	$R^{+}$	24	29	29	25	25	29	29
	$R^{-}$	5	0	0	4	4	0	0
50	$R^{+}$	27	26	29	28	27	26	29
	$R^{-}$	2	3	0	1	2	3	0
100	$R^{+}$	29	27	29	29	29	29	29
	$R^{-}$	0	2	0	0	0	0	0
total	$R^{+}|R^{-}$	80∣7	82∣5	87∣0	82∣5	81∣6	84∣3	87∣0

**Table 7:** Statistical results of TCSD.

Algorithm	Best	Mean	Median	Worst	Std
MRBMO	0.012666	0.012739	0.012714	**0.012984**	0.000070
RBMO	0.012665	0.012759	0.012714	0.013272	0.000147
SOGWO	0.012731	0.013288	0.012879	0.015831	0.000797
LSHADE	0.012669	**0.012735**	0.012707	0.012994	**0.000070**
RIME	0.012948	0.017408	0.017848	0.020430	0.002111
SSA	0.012668	0.013936	0.013201	0.017780	0.001582
LSHADE_SPACMA	**0.012665**	0.012783	**0.012698**	0.013870	0.000276
PSO	0.012675	0.013682	0.013234	0.017455	0.001195
TTHHO	0.012691	0.013689	0.013458	0.016006	0.000863

Bold indicates the minimum value.

**Table 8:** Statistical results of RDP.

Algorithm	Best	Mean	Median	Worst	Std
MRBMO	**2996.34817**	**2996.34838**	**2996.34821**	2996.34930	0.00033
RBMO	2996.34848	2997.47319	2996.35614	3007.42608	3.18454
SOGWO	3019.54252	3037.59170	3038.00575	3055.49922	9.83048
LSHADE	2996.34817	2996.35970	2996.34823	**2996.34836**	**0.00005**
RIME	2998.04641	3020.04269	3016.54626	3061.78099	17.06851
SSA	2996.34839	2996.67139	2996.35137	3005.66973	1.69989
LSHADE_SPACMA	2996.39215	2998.02535	2996.82317	3006.35579	2.80615
PSO	2996.43294	3036.91614	3035.63702	3166.08284	29.64018
TTHHO	3050.14531	4222.52479	4173.65210	5562.22921	1011.74585

Bold indicates the minimum value.

**Table 9:** Statistical results of WBD.

Algorithm	Best	Mean	Median	Worst	Std
MRBMO	0.012666	0.012739	0.012714	**0.012984**	0.000070
RBMO	0.012665	0.012759	0.012714	0.013272	0.000147
SOGWO	0.012731	0.013288	0.012879	0.015831	0.000797
LSHADE	0.012669	**0.012735**	0.012707	0.012994	**0.000070**
RIME	0.012948	0.017408	0.017848	0.020430	0.002111
SSA	0.012668	0.013936	0.013201	0.017780	0.001582
LSHADE_SPACMA	**0.012665**	0.012783	**0.012698**	0.013870	0.000276
PSO	0.012675	0.013682	0.013234	0.017455	0.001195
TTHHO	0.012691	0.013689	0.013458	0.016006	0.000863

Bold indicates the minimum value.

**Table 10:** Statistical results of GTD.

Algorithm	Best	Mean	Median	Worst	Std
MRBMO	2.70086E-12	**2.89217E-10**	**1.16612E-10**	2.35764E-09	**5.20441E-10**
RBMO	2.70086E-12	4.96898E-10	1.16612E-10	**2.05753E-09**	5.79916E-10
SOGWO	2.70086E-12	1.99867E-09	1.36165E-09	2.61397E-08	4.63342E-09
LSHADE	**2.70086E-12**	8.06901E-10	8.88761E-10	2.35764E-09	7.21118E-10
RIME	2.30782E-11	3.98814E-09	2.35764E-09	2.72645E-08	5.85797E-09
SSA	2.30782E-11	1.23977E-08	2.35764E-09	1.76128E-07	3.21443E-08
LSHADE_SPACMA	2.70086E-12	1.56380E-09	1.16612E-10	2.72645E-08	4.92322E-09
PSO	2.70086E-12	5.96709E-09	2.35764E-09	2.72645E-08	8.15863E-09
TTHHO	2.70086E-12	7.91875E-09	2.90473E-09	3.63493E-08	1.00949E-08

Bold indicates the minimum value.

## Data Availability

All data are contained within the article.

## Appendix A. Test Function Suite Details and Results of the Rank Sum Test

**Table A1:** CEC2017 Test function details.

Type	No.	Functions	Minimum Value
Unimodal functions	1	Shifted and Rotated Bent Cigar Function	100
	2	Shifted and Rotated Zakharov Function	200
Simple multimodal functions	3	Shifted and Rotated Rosenbrock’s Function	300
	4	Shifted and Rotated Rastrigin’s Function	400
	5	Shifted and Rotated Expanded Scaffer’s F6 Function	500
	6	Shifted and Rotated Lunacek Bi_Rastrigin Function	600
	7	Shifted and Rotated Non-Continuous Rastrigin’s Function	700
	8	Shifted and Rotated Lecy Function	800
	9	Shifted and Rotated Schwefel’s Function	900
Hybrid functions	10	Hybrid Function 1 (N = 3)	1000
	11	Hybrid Function 2 (N = 3)	1100
	12	Hybrid Function 3 (N = 3)	1200
	13	Hybrid Function 4 (N = 4)	1300
	14	Hybrid Function 5 (N = 4)	1400
	15	Hybrid Function 6 (N = 4)	1500
	16	Hybrid Function 6 (N = 5)	1600
	17	Hybrid Function 6 (N = 5)	1700
	18	Hybrid Function 6 (N = 5)	1800
	19	Hybrid Function 6 (N = 6)	1900
Composition functions	20	Composition Function 1 (N = 3)	2000
	21	Composition Function 2 (N = 3)	2100
	22	Composition Function 3 (N = 4)	2200
	23	Composition Function 4 (N = 4)	2300
	24	Composition Function 5 (N = 5)	2400
	25	Composition Function 6 (N = 5)	2500
	26	Composition Function 7 (N = 6)	2600
	27	Composition Function 7 (N = 6)	2700
	28	Composition Function 9 (N = 3)	2800
	29	Composition Function 10 (N = 3)	2900

**Table A2:** CEC2022 Test function details.

Type	No.	Functions	Minimum Value
Unimodal functions	1	Shifted and full Rotated Zakharov Function	300
Basic functions	2	Shifted and full Rotated Rosenbrock’s Function	400
	3	Shifted and full Rotated Expanded Schaffer’s f6 Function	600
	4	Shifted and full Rotated Non-Continuous Rastrigin’s Function	800
	5	Shifted and full Rotated Levy Function	900
Hybrid functions	6	Hybrid Function 1 (N = 3)	1800
	7	Hybrid Function 2 (N = 6)	2000
	8	Hybrid Function 3 (N = 5)	2200
Composition functions	9	Composition Function 1 (N = 5)	2300
	10	Composition Function 2 (N = 4)	2400
	11	Composition Function 3 (N = 5)	2600
	12	Composition Function 4 (N = 6)	2700

**Table A3:** The results of Wilcoxon rank sum test *p*-value on CEC2017 when D = 30.

Function	RBMO	SOGWO	RIME	SSA	LSHADE_SPACMA	PSO	TTHHO
F1	9.211E-05	3.020E-11	3.020E-11	**5.692E-01**	2.195E-08	3.020E-11	3.020E-11
F2	2.499E-03	3.020E-11	3.020E-11	3.020E-11	3.020E-11	3.020E-11	3.020E-11
F3	5.264E-04	6.722E-10	3.010E-07	**3.042E-01**	**7.245E-02**	1.430E-05	3.020E-11
F4	**2.838E-01**	5.092E-08	3.474E-10	3.020E-11	8.564E-04	1.429E-08	3.020E-11
F5	8.663E-05	3.338E-11	3.690E-11	3.020E-11	1.850E-08	3.020E-11	3.020E-11
F6	2.416E-02	4.077E-11	6.066E-11	3.020E-11	3.965E-08	3.690E-11	3.020E-11
F7	2.510E-02	1.254E-07	2.371E-10	3.020E-11	1.106E-04	6.722E-10	3.020E-11
F8	1.873E-07	4.504E-11	3.020E-11	3.020E-11	2.371E-10	8.993E-11	3.020E-11
F9	2.388E-04	6.669E-03	2.236E-02	7.119E-09	3.501E-03	3.778E-02	3.338E-11
F10	1.174E-03	3.020E-11	8.993E-11	8.993E-11	2.879E-06	9.260E-09	3.020E-11
F11	**9.049E-02**	3.020E-11	3.020E-11	7.389E-11	6.526E-07	3.020E-11	3.020E-11
F12	**7.483E-02**	3.020E-11	3.020E-11	3.592E-05	8.315E-03	2.959E-05	3.020E-11
F13	1.606E-06	3.020E-11	3.020E-11	3.020E-11	4.077E-11	3.020E-11	3.020E-11
F14	9.514E-06	3.020E-11	5.573E-10	5.494E-11	5.092E-08	5.072E-10	3.020E-11
F15	7.288E-03	4.118E-06	3.352E-08	6.518E-09	4.744E-06	4.943E-05	3.690E-11
F16	1.175E-04	3.368E-05	7.119E-09	1.957E-10	9.883E-03	6.010E-08	3.020E-11
F17	**3.403E-01**	3.020E-11	3.020E-11	3.020E-11	3.338E-11	3.020E-11	3.020E-11
F18	4.639E-05	3.020E-11	3.020E-11	3.690E-11	4.444E-07	2.371E-10	3.020E-11
F19	3.339E-03	1.529E-05	3.352E-08	2.669E-09	2.783E-07	1.988E-02	2.371E-10
F20	**2.772E-01**	4.118E-06	3.825E-09	3.020E-11	2.052E-03	1.102E-08	5.573E-10
F21	7.695E-08	7.380E-10	2.196E-07	2.154E-10	6.528E-08	1.174E-09	4.504E-11
F22	4.353E-05	2.317E-06	3.820E-10	3.338E-11	**1.297E-01**	6.066E-11	3.020E-11
F23	3.965E-08	2.572E-07	4.975E-11	3.020E-11	5.971E-05	3.020E-11	3.020E-11
F24	1.174E-03	1.777E-10	3.197E-09	**9.941E-01**	1.302E-03	1.028E-06	3.020E-11
F25	5.264E-04	6.526E-07	2.154E-10	5.092E-08	**6.787E-02**	5.561E-04	3.820E-10
F26	1.765E-02	1.547E-09	9.533E-07	4.573E-09	4.639E-05	1.857E-09	3.020E-11
F27	1.857E-09	3.020E-11	9.919E-11	3.034E-03	7.043E-07	9.756E-10	3.020E-11
F28	3.835E-06	2.154E-06	3.820E-10	6.066E-11	**6.520E-01**	1.493E-04	3.020E-11
F29	1.680E-03	3.020E-11	3.020E-11	**9.334E-02**	3.848E-03	7.599E-07	3.020E-11

Bold indicates values less than 0.05.

**Table A4:** The results of Wilcoxon rank sum test *p*-value on CEC2017 when D = 50.

Function	RBMO	SOGWO	RIME	SSA	LSHADE_SPACMA	PSO	TTHHO
F1	3.020E-11	3.020E-11	3.020E-11	3.020E-11	3.020E-11	3.020E-11	3.020E-11
F2	**9.049E-02**	3.020E-11	3.020E-11	3.020E-11	4.504E-11	3.020E-11	3.020E-11
F3	1.698E-08	3.020E-11	4.975E-11	4.676E-02	2.610E-10	7.389E-11	3.020E-11
F4	5.092E-08	5.494E-11	3.690E-11	3.020E-11	2.439E-09	6.066E-11	3.020E-11
F5	1.407E-04	3.338E-11	3.020E-11	3.020E-11	3.324E-06	3.020E-11	3.020E-11
F6	1.311E-08	3.020E-11	3.020E-11	3.020E-11	5.494E-11	3.020E-11	3.020E-11
F7	2.377E-07	2.610E-10	1.411E-09	3.020E-11	6.010E-08	3.690E-11	3.020E-11
F8	2.133E-05	3.020E-11	3.020E-11	3.020E-11	1.957E-10	3.020E-11	3.020E-11
F9	9.833E-08	**9.926E-02**	2.236E-02	2.380E-03	7.695E-08	**1.154E-01**	3.690E-11
F10	2.371E-10	3.020E-11	3.020E-11	7.119E-09	3.020E-11	3.020E-11	3.020E-11
F11	4.573E-09	3.020E-11	3.020E-11	6.722E-10	1.287E-09	3.020E-11	3.020E-11
F12	5.462E-06	3.020E-11	3.020E-11	3.497E-09	3.256E-07	1.777E-10	3.020E-11
F13	2.126E-04	3.020E-11	3.020E-11	3.020E-11	3.020E-11	3.020E-11	3.020E-11
F14	4.515E-02	3.020E-11	3.020E-11	7.739E-06	**1.120E-01**	**7.483E-02**	3.020E-11
F15	6.203E-04	**2.062E-01**	7.043E-07	4.183E-09	1.175E-04	1.441E-02	3.020E-11
F16	3.183E-03	**1.809E-01**	7.695E-08	1.102E-08	6.913E-04	6.377E-03	5.494E-11
F17	**6.414E-01**	3.020E-11	3.020E-11	5.494E-11	1.337E-05	7.389E-11	3.020E-11
F18	4.825E-01	3.020E-11	3.020E-11	**3.953E-01**	**6.952E-01**	1.988E-02	3.020E-11
F19	2.499E-03	3.917E-02	6.765E-05	9.756E-10	3.159E-10	8.120E-04	2.227E-09
F20	1.606E-06	3.820E-10	1.464E-10	3.020E-11	2.015E-08	3.690E-11	3.020E-11
F21	3.825E-09	2.755E-03	4.676E-02	4.714E-04	1.254E-07	**5.369E-02**	3.020E-11
F22	1.957E-10	2.371E-10	1.206E-10	3.020E-11	3.825E-09	3.020E-11	3.020E-11
F23	1.070E-09	2.439E-09	2.154E-10	3.020E-11	7.773E-09	3.020E-11	3.020E-11
F24	1.102E-08	3.020E-11	1.850E-08	4.427E-03	1.287E-09	8.891E-10	3.020E-11
F25	1.529E-05	3.474E-10	5.494E-11	1.442E-03	6.518E-09	8.663E-05	3.020E-11
F26	2.491E-06	4.077E-11	5.072E-10	2.371E-10	1.102E-08	2.154E-10	3.020E-11
F27	8.993E-11	3.020E-11	2.610E-10	8.883E-06	1.957E-10	1.957E-10	3.020E-11
F28	7.599E-07	3.159E-10	4.077E-11	5.494E-11	1.171E-02	2.669E-09	3.020E-11
F29	6.528E-08	3.020E-11	3.020E-11	3.848E-03	1.777E-10	5.967E-09	3.020E-11

Bold indicates values less than 0.05.

**Table A5:** The results of Wilcoxon rank sum test *p*-value on CEC2017 when D = 100.

Function	RBMO	SOGWO	RIME	SSA	LSHADE_SPACMA	PSO	TTHHO
F1	3.020E-11	3.020E-11	3.020E-11	3.020E-11	3.020E-11	3.020E-11	3.020E-11
F2	4.119E-01	3.020E-11	3.020E-11	3.020E-11	9.833E-08	3.020E-11	6.696E-11
F3	3.020E-11	3.020E-11	4.077E-11	1.857E-09	3.020E-11	3.020E-11	3.020E-11
F4	1.202E-08	8.993E-11	8.993E-11	3.020E-11	1.464E-10	3.020E-11	3.020E-11
F5	3.520E-07	6.696E-11	3.338E-11	3.020E-11	1.892E-04	3.020E-11	3.020E-11
F6	3.197E-09	3.020E-11	3.690E-11	3.020E-11	3.338E-11	3.020E-11	3.020E-11
F7	4.504E-11	3.020E-11	3.020E-11	3.020E-11	7.389E-11	3.020E-11	3.020E-11
F8	3.256E-07	3.020E-11	3.020E-11	3.020E-11	7.389E-11	3.020E-11	3.020E-11
F9	1.957E-10	**1.624E-01**	1.383E-02	3.835E-06	7.389E-11	2.624E-03	3.020E-11
F10	1.329E-10	3.020E-11	3.020E-11	3.020E-11	3.020E-11	3.020E-11	3.020E-11
F11	3.020E-11	3.020E-11	3.020E-11	3.020E-11	3.020E-11	3.020E-11	3.020E-11
F12	3.020E-11	3.020E-11	3.020E-11	3.020E-11	3.020E-11	3.020E-11	3.020E-11
F13	9.587E-01	3.020E-11	3.020E-11	2.610E-10	9.533E-07	4.077E-11	3.020E-11
F14	3.831E-05	3.020E-11	3.020E-11	2.922E-09	4.200E-10	4.504E-11	3.020E-11
F15	9.533E-07	8.841E-07	1.613E-10	8.663E-05	7.088E-08	8.663E-05	3.020E-11
F16	1.492E-06	5.570E-03	1.311E-08	3.825E-09	3.835E-06	5.573E-10	3.020E-11
F17	4.637E-03	4.077E-11	3.020E-11	3.010E-07	2.068E-02	3.020E-11	3.020E-11
F18	5.555E-02	3.020E-11	3.020E-11	3.770E-04	1.206E-10	3.020E-11	3.020E-11
F19	4.207E-02	1.988E-02	5.607E-05	2.390E-08	4.504E-11	5.607E-05	7.389E-11
F20	3.338E-11	3.690E-11	3.338E-11	3.020E-11	3.020E-11	3.020E-11	3.020E-11
F21	3.646E-08	**9.470E-01**	3.147E-02	3.671E-03	4.077E-11	1.635E-05	3.020E-11
F22	4.077E-11	1.957E-10	7.389E-11	3.020E-11	2.610E-10	3.020E-11	3.020E-11
F23	3.020E-11	3.020E-11	3.690E-11	3.020E-11	3.020E-11	3.020E-11	3.020E-11
F24	3.020E-11	3.020E-11	4.504E-11	3.825E-09	3.020E-11	3.020E-11	3.020E-11
F25	9.063E-08	1.411E-09	5.462E-09	9.833E-08	9.756E-10	2.371E-10	3.020E-11
F26	2.254E-04	3.020E-11	3.338E-11	3.835E-06	4.975E-11	4.077E-11	3.020E-11
F27	3.020E-11	3.020E-11	4.077E-11	1.429E-08	3.020E-11	4.077E-11	3.020E-11
F28	5.600E-07	1.613E-10	3.020E-11	2.597E-05	7.599E-07	3.010E-07	3.020E-11
F29	3.020E-11	3.020E-11	3.020E-11	3.020E-11	3.020E-11	3.020E-11	3.020E-11

Bold indicates values less than 0.05.

## Appendix B. Engineering Application Design Issues

### Appendix B.1. Extension/Compression Spring Problem

Minimize: $f\left(\vec{x}\right)=\left(x_{3}+2\right)x_{2}x_{1}^{2}$

Subject to: $\begin{array}{c} g_{1}\left(\vec{x}\right)=1-\frac{x_{2}^{3}x_{3}}{71785x_{1}^{4}}\leq 0\\ g_{2}\left(\vec{x}\right)=\frac{4x_{2}^{2}-x_{1}x_{2}}{12566(x_{2}x_{1}^{3}-x_{1}^{4})}+\frac{1}{5108x_{1}^{2}}\leq 0\\ g_{3}\left(\vec{x}\right)=1-\frac{140.45x_{1}}{x_{2}^{2}x_{3}}\leq 0\\ g_{4}\left(\vec{x}\right)=\frac{x_{1}+x_{2}}{1.5}-1\leq 0\\ 0.05\leq x_{1}\leq 2,0.25\leq x_{2}\leq 1.3,2\leq x_{3}\leq 15. \end{array}$

### Appendix B.2. Reducer Design Problem

Minimize: $f\left(\vec{x}\right)=0.7854x_{1}x_{2}^{2}\left(3.3333x_{3}^{2}+14.9334x_{3}-43.0934\right)-1.508x_{1}\left(x_{6}^{2}+x_{7}^{2}\right)+7.4777\left(x_{6}^{3}+x_{7}^{3}\right)$

Subject to: $\begin{array}{c} g_{1}\left(\vec{x}\right)=\frac{27}{x_{1}x_{2}^{2}x_{3}}-1\leq 0,\\ g_{2}\left(\vec{x}\right)=\frac{397.5}{x_{1}x_{2}^{2}x_{3}^{2}}-1\leq 0,\\ g_{3}\left(\vec{x}\right)=\frac{1.93x_{4}^{3}}{x_{2}x_{3}x_{6}^{4}}-1\leq 0,\\ g_{4}\left(\vec{x}\right)=\frac{1.93x_{5}^{3}}{x_{2}x_{3}x_{7}^{4}}-1\leq 0,\\ g_{5}\left(\vec{x}\right)=\frac{\sqrt{{\left(\frac{745x_{4}}{x_{2}x_{3}}\right)}^{2}+16.9\times 10^{6}}}{110.0x_{6}^{3}}-1\leq 0,\\ g_{6}\left(\vec{x}\right)=\frac{\sqrt{{\left(\frac{745x_{4}}{x_{2}x_{3}}\right)}^{2}+157.5\times 10^{6}}}{85.0x_{6}^{3}}-1\leq 0,\\ g_{7}\left(\vec{x}\right)=\frac{x_{2}x_{3}}{40}-1\leq 0,\\ g_{8}\left(\vec{x}\right)=\frac{5x_{2}}{x_{1}}-1\leq 0,\\ g_{9}\left(\vec{x}\right)=\frac{x_{1}}{12x_{2}}-1\leq 0,\\ g_{10}\left(\vec{x}\right)=\frac{1.5x_{6}+1.9}{x_{4}}-1\leq 0,\\ g_{11}\left(\vec{x}\right)=\frac{1.1x_{7}+1.9}{x_{5}}-1\leq 0,\\ 2.6\leq x_{1}\leq 3.6,\\ 0.7\leq x_{2}\leq 0.8,\\ 17\leq x_{3}\leq 28,\\ 7.3\leq x_{4}\leq 8.3,\\ 7.8\leq x_{5}\leq 8.3,\\ 2.9\leq x_{6}\leq 3.9,\\ 5.0\leq x_{7}\leq 5.5, \end{array}$

### Appendix B.3. Welded Beam Problem

Minimize: $f\left(\vec{x}\right)=1.10471x_{1}^{2}x_{2}+0.04811x_{3}x_{4}\left(14.0+x_{2}\right)$

Subject to: $\begin{array}{c} g_{1}\left(\vec{x}\right)=\tau \left(\vec{x}\right)-\tau_{\max}\leq 0\\ g_{2}\left(\vec{x}\right)=\sigma \left(\vec{x}\right)-\tau_{\max}\leq 0\\ g_{3}\left(\vec{x}\right)=\delta \left(\vec{x}\right)-\tau_{\max}\leq 0\\ g_{4}\left(\vec{x}\right)=x_{1}-x_{4}\leq 0\\ g_{5}\left(\vec{x}\right)=P-P_{c}\left(\vec{x}\right)\leq 0\\ g_{6}\left(\vec{x}\right)=0.125-x_{1}\leq 0\\ g_{7}\left(\vec{x}\right)=1.10471x_{1}^{2}+0.04811x_{3}x_{4}\left(14.0+x_{2}\right)-5.0\leq 0\\ 0.1\leq x_{1},x_{4}\leq 2,0.1\leq x_{2},x_{3}\leq 10 \end{array}$

where: $\begin{array}{c} \tau \left(\vec{x}\right)=\sqrt{{\left(\tau^{\prime }\right)}^{2}+2\tau^{\prime }\tau^{\prime \prime }\frac{x_{2}}{2R}+{\left(\tau^{\prime }\right)}^{2}},\\ \tau^{\prime }=\frac{P}{\sqrt{2x_{1}x_{2}}},\tau^{\prime \prime }=\frac{MR}{J},\\ M=P(L+\frac{x_{2}}{2}),\\ R=\sqrt{\frac{x_{2}^{2}}{4}+{\left(\frac{x_{1}+x_{3}}{2}\right)}^{2}},\\ J=2(\sqrt{2}x_{1}x_{2}\left(\frac{x_{2}^{2}}{12}+{\left(\frac{x_{1}+x_{3}}{2}\right)}^{2}\right)),\\ \sigma \left(\vec{x}\right)=\frac{6PL}{x_{4}x_{3}^{2}},\delta \left(x\right)=\frac{4PL^{3}}{Ex_{3}^{3}x_{4}},\\ P_{c}\left(x\right)=\frac{4.013E\sqrt{\frac{x_{3}^{2}x_{4}^{6}}{36}}}{L^{2}}\left(1-\frac{x_{3}}{2L}\sqrt{\frac{E}{4G}}\right),\\ P=6000lb,L=14in,\delta_{\max}=0.25in,\\ E=30\times 10^{6}psi,G=12\times 10^{6}psi,\\ \tau_{\max}=13600psi,\sigma_{\max}=30000psi. \end{array}$

### Appendix B.4. Gear Train Design Problem

Minimize: $f\left(\vec{x}\right)={\left(\frac{1}{6.931}-\frac{x_{2}x_{3}}{x_{1}x_{4}}\right)}^{2}$

Subject to: $12\leq x_{i}\leq 60,i=1,2,3,4.$
